# Macrophage NFATC2 mediates angiogenic signaling during mycobacterial infection

**DOI:** 10.1016/j.celrep.2022.111817

**Published:** 2022-12-13

**Authors:** W. Jared Brewer, Ana María Xet-Mull, Anne Yu, Mollie I. Sweeney, Eric M. Walton, David M. Tobin

**Affiliations:** 1 Department of Molecular Genetics & Microbiology, Duke University School of Medicine, Durham, NC 27710, USA; 2 Department of Immunology, Duke University School of Medicine, Durham, NC 27710, USA; 3 Lead contact

## Abstract

During mycobacterial infections, pathogenic mycobacteria manipulate both host immune and stromal cells to establish and maintain a productive infection. In humans, non-human primates, and zebrafish models of infection, pathogenic mycobacteria produce and modify the specialized lipid trehalose 6,6′-dimycolate (TDM) in the bacterial cell envelope to drive host angiogenesis toward the site of forming granulomas, leading to enhanced bacterial growth. Here, we use the zebrafish-*Mycobacterium marinum* infection model to define the signaling basis of the host angiogenic response. Through intravital imaging and cell-restricted peptide-mediated inhibition, we identify macrophage-specific activation of NFAT signaling as essential to TDM-mediated angiogenesis *in vivo*. Exposure of cultured human cells to *Mycobacterium tuberculosis* results in robust induction of VEGFA, which is dependent on a signaling pathway downstream of host TDM detection and culminates in NFATC2 activation. As granuloma-associated angiogenesis is known to serve bacterial-beneficial roles, these findings identify potential host targets to improve tuberculosis disease outcomes.

## In brief

*Mycobacterium tuberculosis* infection in humans results in the growth of blood vessels at forming granulomas via engagement with the bacterial glycolipid trehalose 6,6′-dimycolate. Brewer et al. identify macrophage-specific activation of NFAT signaling as a key step in macrophage VEGFA production during mycobacterial infection and angiogenesis, suggesting therapeutic targets for tuberculosis.

## Graphical Abstract

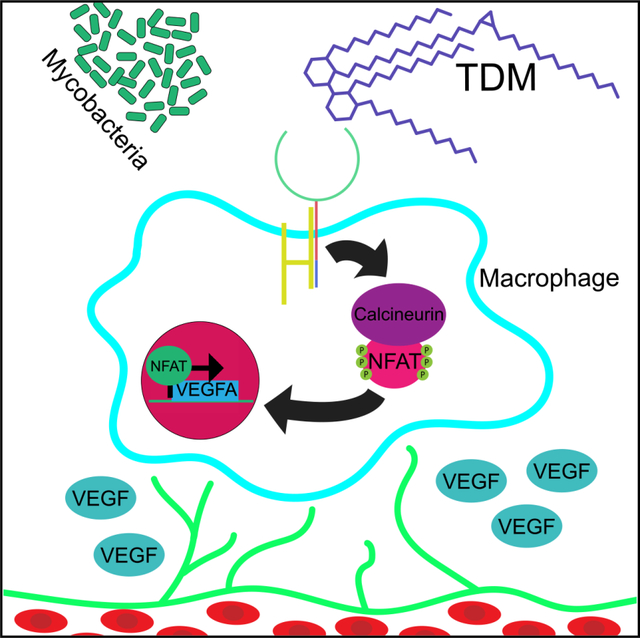

## INTRODUCTION

The host immune response to infection is driven by an intricately regulated, but occasionally discordant or maladaptive, immune response to pathogenic stimuli at the cell-intrinsic, innate, and adaptive levels.^1^ While the contributions of immune cells have been widely studied, there is growing appreciation that non-immune populations, including stromal cells and the endothelium,^2–4^ are also crucial in shaping the host response during both acute and chronic infections.^5–7^ Just as pathogens have evolved sophisticated mechanisms to hijack signaling pathways in immune cells,^8^ they have also been shown to manipulate developmental and homeostatic processes to direct them toward pathogen-beneficial host responses.^9,10^

*Mycobacterium tuberculosis* (*Mtb*) is among history’s most widespread and successful pathogens. It has evolved a range of sophisticated mechanisms to manipulate its human host in order to survive, replicate, and transmit. Upon infection, *Mtb* induces a complex immune response wherein innate immune cells, initially consisting primarily of macrophages, congregate at the bacterial focus and then undergo an epithelioid transformation and interdigitate to form a granuloma, the hallmark feature of tuberculosis (TB), which provides both a replicative niche and a primary host-pathogen interface of TB disease.^11–13^ Granuloma-associated vasculature has long been noted in human and animal models of TB,^14,15^ but the mechanisms of induction and precise contributions to infection are not yet fully understood.

Many of the major pathological features of mycobacterial granulomas, including associated vascularization, are conserved from zebrafish to humans.^16,17^ Zebrafish can be infected with a natural pathogen, *Mycobacterium marinum*, which induces a robust angiogenic response during granuloma formation. This process, much like that in humans, non-human primates, and rabbits, is associated with production of the pro-angiogenic chemokine, Vegfaa, at the site of infection.^18^ This chemokine is a critical regulator of angiogenesis in both developmental and pathological contexts, and production of Vegfa has also been probed in murine granuloma models.^19^ Similarly, human granulomas have been shown to express VEGFA and are physically associated with blood vessels that associate with the outer granuloma layers.^20^ Subsequent work has demonstrated a role for these vessels in supporting bacterial growth and in dissemination of the bacilli from their primary site of infection.^21^ Recent profiling of human and non-human primate granulomas has confirmed the presence of aberrant vasculature associated with *Mtb* granulomas^22,23^ in the context of a non-canonical type 2 immune response.^13^

Pathogenic mycobacteria have evolved specialized mechanisms to promote and accelerate angiogenesis. Notably, the extensively modified and essential outer cell envelope component trehalose 6,6′-dimycolate (TDM) is *cis*-cyclopropanated by the pathogen-specific enzyme PcaA.^24^ Mutation of *pcaA* results in a reduction in granuloma angiogenesis and reduction in bacterial burden; correspondingly, cyclopropanated TDM alone is sufficient to induce host angiogenesis.^25–27^ As *pcaA*-dependent vascularization supports bacterial growth, factors driving this represent potential sites of therapeutic intervention, yet the signals that mediate this host process remain unclear.

TDM is an extraordinarily long-chain, hydrophobic (C_60_-C_90_) glycolipid that has been shown to be detected in cell culture and murine models by host C-type lectin receptors, most notably MCL (*CLEC4D*) and MINCLE (*CLEC4E*), as well as by Toll-like receptor 2 (TLR2), CD14, and MARCO.^28–30^ Canonically, C-type lectin signaling is transmitted through a CARD9-NF-κB signaling pathway that results in the transcription and production of TNF-α, IL-1β, IL-6, and other cytokines.^31–35^ However, beyond CARD9, a number of other downstream signaling pathways are engaged by C-type lectin activation and likely control discrete aspects of immune signaling.^35,36^

Here, we synthesize findings from zebrafish and cell culture models to define the *in vivo* angiogenic response induced by pathogenic mycobacteria. Contrary to classical models of C-type lectin signaling, we find that *cis*-cyclopropanated TDM exerts its pro-angiogenic effects through an alternative NFAT-driven pathway rather than canonical CARD9-NF-κB signaling. We use peptide-mediated, cell-specific inhibition of NFAT to demonstrate that both early and mature granuloma angiogenesis are dependent upon macrophage-NFAT signaling. We identify Nfatc2a as the predominant isoform mediating *vegfaa* induction and angiogenesis. These findings define a basis for granuloma-associated angiogenesis during pathogenic mycobacterial infections and suggest targets for host-directed therapeutic interventions during TB.

## RESULTS

### Macrophage induction of *vegfaa* and angiogenesis during mycobacterial infection

**Figure 1.**
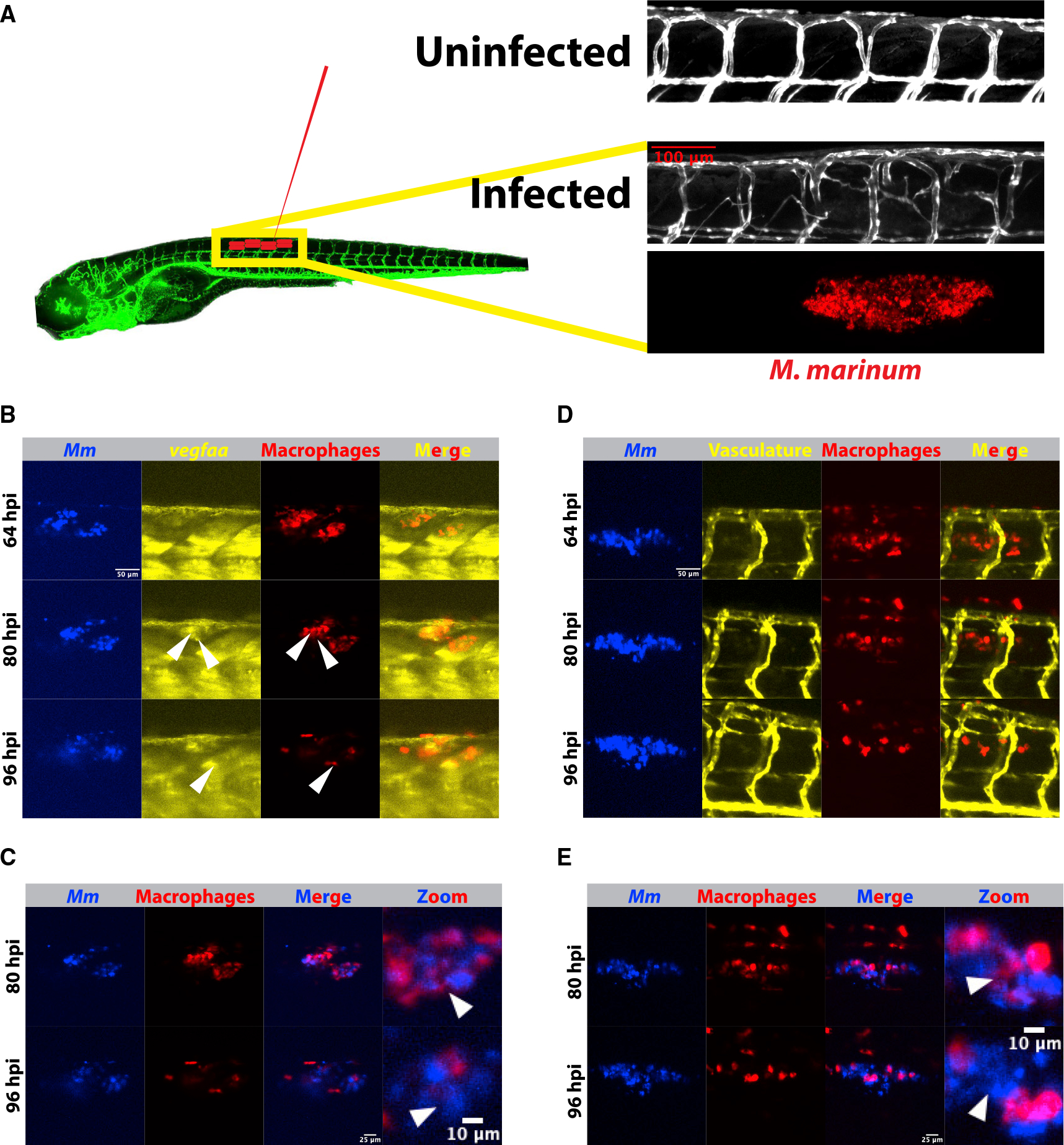
Kinetics of macrophage *vegfaa* induction and angiogenesis (A) Schematic depicting Tg(*kdrl*:*eGFP*^*s843*^) zebrafish and the approximate injection site, where ~50–150 fluorescent *Mycobacterium marinum* are injected. Inset above displays a confocal image from the trunk region of a *kdrl*:*eGFP* zebrafish larva and the highly stereotyped intersomitic vascular patterning. Below, an image from an infected 4 days post infection (dpi) larval zebrafish showing the aberrant vasculature that develops over the course of infection and is the subject of the later quantitation. Also shown is the bacterial channel, demonstrating that this angiogenesis occurs at the site of infection. (B) Time lapse of larval Tg(*irg1*:*tdTomato*^*xt40*^); TgBAC*(vegfaa*:*eGFP*^*pd260*^) zebrafish infected with 50–150 *Mm*-*eBFP2* (blue) showing an increase in *vegfaa* reporter (yellow) levels in macrophages (red) over 96 h of imaging. Arrowheads indicate macrophages positive for *vegfaa*:*egfp* expression. Scale bar, 50 μm. Representative of 32 fish from one experimental replicate. Stills from Video S1. (C) Overlay of macrophage and bacterial fluorescence channels from (B) shows a general increase in the extracellular bacterial growth beginning approximately 80 h post infection, correlating with the increasing *vegfaa* signal in (B). Scale bars, 25 μm and 10 μm (in cropped images). Arrowheads indicate examples of intracellular bacteria at 80 hpi and then extracellular bacteria at the same approximate location at 96 hpi. (D) Time-lapse imaging of zebrafish vasculature and macrophages using the transgenic line *irg1*:*tdTomato*; *kdrl*:*eGFP*. Angiogenesis accelerates as extracellular bacteria accumulate, but at a relative delay from induction of the *vegfaa*:*eGFP* reporter (64–80 hpi versus 80–96 hpi), suggesting that *vegfaa* transcription precedes the angiogenic response. Representative of 32 fish from one experimental replicate. Scale bar, 50 μm. Stills from Video S2. (E) Overlay of the macrophage and bacterial fluorescence channels showing increasing extracellular growth, with the highest degree of angiogenesis at time points with the largest number of extracellular bacteria (D). Scale bars, 25 μm and 10 μm (in cropped images).

Injection of live *Mycobacterium marinum* into the dorsal trunk of the zebrafish larva is sufficient to induce a robust angiogenic response adjacent to nascent granulomas in a macrophage-dependent manner^18^ (Figure 1A). The stereotyped vasculature along this region of the larva allows facile quantitation of neovascularization during and after granuloma formation or other insult.^37–40^ We have previously demonstrated that *cis*-cyclopropanated TDM is required for the induction of *vegfaa* and angiogenesis at the site of infection. Furthermore, we found that genetic blockade of Vegfaa signaling was sufficient to abolish angiogenesis during infection with wild-type mycobacteria.^25^ Taken together, these findings suggest that the failure to induce *vegfaa* is a major contributor to the loss of angiogenesis in *pcaA*-deficient granulomas.

To study this phenomenon further, we began by examining the kinetics of *vegfaa* induction to identify the cellular source of *vegfaa* during granuloma formation. To test whether macrophages were a significant source of *vegfaa*, we developed a macrophage-specific reporter using the previously described *acod1* promoter (also known as *irg1*), Tg(*irg1*:*tdTomato*^*xt40*^) (from here, *irg1*:*tdTomato*). *irg1* has been found to be expressed specifically in zebrafish macrophages and is upregulated during infection.^41,42^ We then crossed this line with the *vegfaa* reporter line TgBAC(*vegfaa*:*eGFP*^*pd260*^)^43^ (*vegfaa*:*eGFP* throughout) and infected double transgenic *irg1*:*tdTomato*; *vegfaa*:*eGFP* progeny with *M*. *marinum* expressing eBFP2 (*Mm*-eBFP2) to simultaneously visualize bacteria, macrophage localization, and *vegfaa* production *in vivo*.^44^

We began imaging at a time point that preceded robust induction of *vegfaa*:*eGFP* but would allow us to capture the maximum time span of these events. We observed an increase in *vegfaa* reporter signal over time that appeared largely localized to macrophages (Figure 1B). We observed that bacteria initially grew primarily intracellularly within individual macrophages at 36 h post infection but began to grow in characteristic extracellular cords by approximately 84 h post infection with little to no intracellular containment at this site by 96 h post infection (Figure 1C). The increase in extracellular growth coincided with the induction of an eGFP signal in macrophages at ~64 h (Figure 1B), suggesting that, at low overall burden, intracellular detection does not induce *vegfaa* expression, while extracellular engagement correlates with *vegfaa* expression during early stages of granuloma formation (Figure 1B; Video S1).

We next visualized the production of angiogenic vessels throughout infection in parallel to our characterization of *vegfaa* induction. Due to an inability to separate discrete emission wave-lengths using two GFP reporter lines, we were unable to examine all four components (bacteria, *vegfaa* induction, macrophages, and vasculature) simultaneously. To relate this process directly to the angiogenesis observed in mycobacterial granulomas, we crossed the *irg1*:*tdTomato* macrophage reporter to the Tg(*kdrl*:*eGFP*^*s843*^) (from here, *kdrl*:*eGFP*) line, which labels vasculature (*irg1*:*tdTomato*; *kdrl*:*eGFP*). Under the same conditions and burden at which we infected the *vegfaa* and macrophage dual reporter line, we observed robust vascularization at approximately 96 h post-infection, subsequent to initial granuloma formation and *vegfaa* induction (Figures 1C–1E; Video S2).

### Genetic *card9* deficiency does not compromise mycobacteria-induced angiogenesis

Given these observations suggesting that macrophages engaging extracellular bacteria are an important source of *vegfaa* expression, we interrogated pattern recognition receptor (PRR) signaling pathways that had been implicated in host responses to TDM, a major external component of the mycobacterial cell envelope. We had previously found that *myd88* was dispensable for the induction of angiogenesis in response to TDM *in vivo*.^25,28^ This suggested that the described TLR2-mediated responses that function downstream of TDM detection in some contexts were unlikely to be required for this process. Rather, we found that the FcgR homologs in zebrafish, *fcer1g* and *fcer1gl*, are required for the full angiogenic response to TDM,^25^ implicating MINCLE/MCL-like C-type lectin receptor signaling in mediating this response.^34,45^

**Figure 2.**
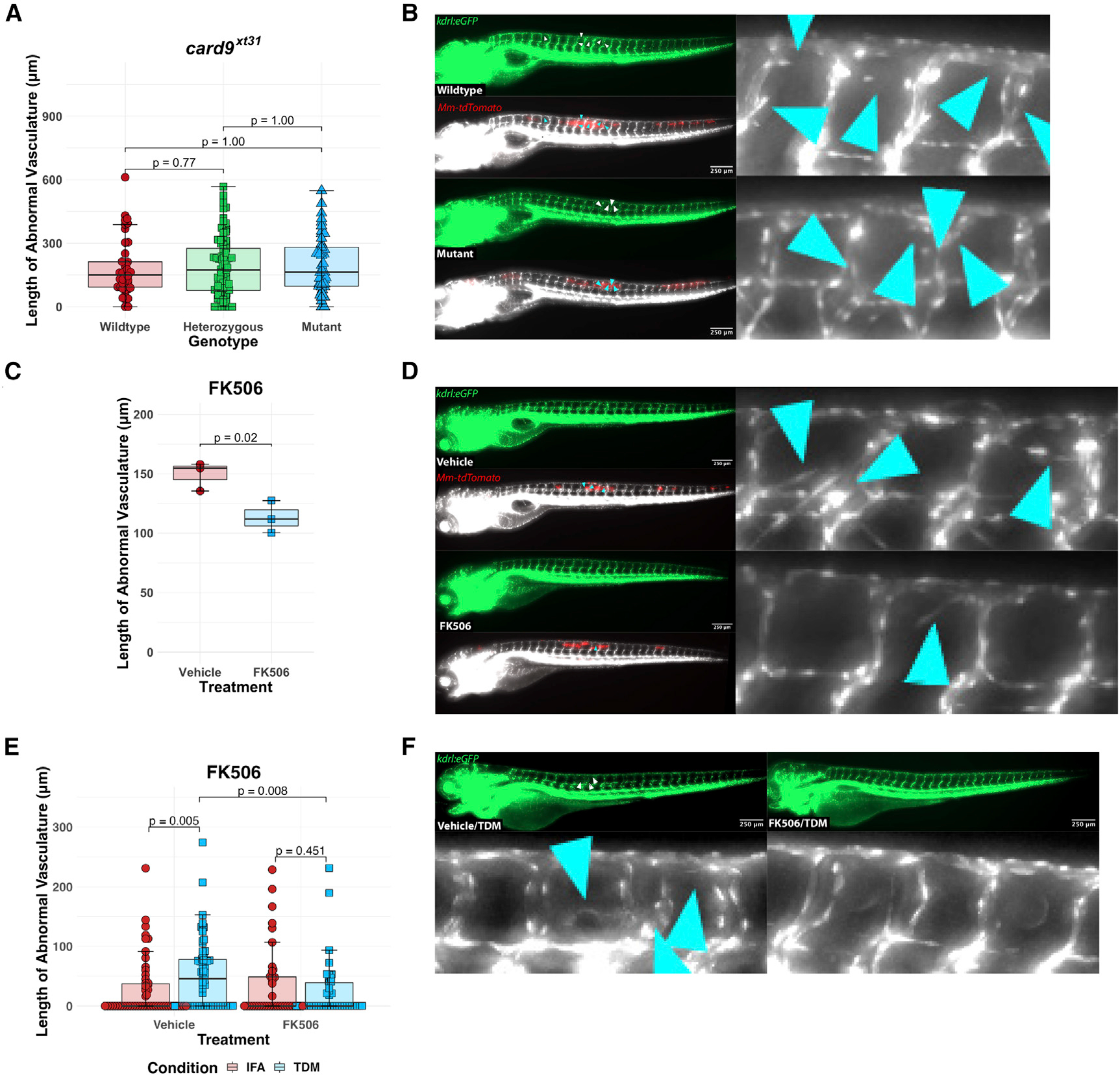
Pharmacological blockade of NFAT signaling inhibits angiogenesis while genetic ablation of *card9* does not (A) Quantitation of angiogenic vessels along the trunk of *card9* mutant, heterozygous, and wild-type animals 4 days post infection (dpi) with *Mm*-tdTomato. Each data point represents a single larva, n = 44 wild-type, 89 heterozygotes, 45 homozygous mutants. No statistically significant differences observed across the groups by Dunn’s Kruskal-Wallis multiple comparisons test with Holm error correction. Representative of three independent experiments. Additional independent replicates provided in Figures S1B and S1C. (B) Representative images from *card9* wild-type and homozygous mutant zebrafish. Note the emergence of non-stereotypical vessels into the somites near the site of infection (inset). Arrowheads indicate regions of neovascularization. Scale bar, 250 μm. (C) Quantitation of angiogenesis along the trunk of *Mm*-tdTomato-infected wild-type *kdrl*:*eGFP* zebrafish treated with either 125 nM FK506 or 0.0125% DMSO (vehicle) diluted in E3 medium. Each point represents mean vessel length within an independent biological replicate of 60–96 animals, with equal numbers of animals per replicate across three replicates. Box-and-whisker plot shows interquartile ranges. Statistical significance determined by Student’s t test. (D) Representative images of FK506- or vehicle-treated larvae. A single dose at a concentration that showed no developmental toxicity was provided immediately after infection. Doses at or above ~500 nM proved developmentally toxic. Arrowheads indicate ectopic vessels at the site of infection. (E) Quantitation of angiogenesis from wild-type *kdrl*:*eGFP* larval zebrafish injected with 2 mg/mL TDM in ~10–20 nL bolus or comparable volume of IFA vehicle alone. Treatment with 125 nM FK506 or 0.0125% ethanol (vehicle) for 2 dpi. Points shown are a random subset of 200 fish (50 fish per group) out of a total of 524 represented larvae to minimize data crowding. Each point represents a single larva with data pooled from three independent experiments. Statistics from Dunn’s Kruskal-Wallis multiple comparisons test with Holm error correction on the whole dataset. (F) Representative images from FK506-treated or ethanol (vehicle)-treated larvae at 2 dpi of TDM-injected larvae. FK506-treated larvae demonstrate a reduction in the degree of angiogenesis compared with the ethanol-treated group. Arrowheads indicate regions of angiogenesis.

As many of the downstream activities of C-type lectin receptors have been ascribed to the activation of CARD9-NF-κB signaling,^32–35,46^ we assessed what role this pathway might play in angiogenesis during mycobacterial infection. We developed a *card9* knockout zebrafish line using CRISPR-Cas9 that carries a 28 bp insertion, resulting in an early stop codon after 59 amino acids (*card9*^*xt31*^) (Figure S1A). We then assayed these animals in the *kdrl*:*eGFP* transgenic background by incrossing *kdrl*:*eGFP*; *card9*^*xt31/+*^ animals and infecting the resulting offspring with tdTomato-fluorescent *M*. *marinum* (*Mm*-tdTomato) at 2 days post fertilization (dpf)^18,39^ (Figures 2A and 2B). We quantitated the resulting aberrant vasculature at 4 days post infection (dpi) under genotypic blinding and post hoc matched these measurements to genotype. There were no significant differences between the three genotypes (Figures S1B and S1C), suggesting either redundancy between multiple established pathways or the existence of an alternative pathway downstream of TDM detection that was *fcer1g/fcer1gl* dependent, but independent of both *myd88* and *card9*.

### Pharmacological inhibition of NFAT induction limits mycobacteria-induced angiogenesis

Although many of the physiological consequences of C-type lectin receptor induction are often ascribed to CARD9-NF-κB signaling, this PRR class is also known to activate a distinct transcription factor family with known roles in immunity—the nuclear factor of activated T cells or NFAT.^35,36^ This calcium-responsive transcription factor pathway is best described in its role regulating T cell biology, but there are numerous reports describing various roles for the members of this pathway in other cell types, including macrophages.^47–51^ Given that there are four mammalian members of this pathway and six zebrafish homologs with potentially overlapping functions, we began with a pharmacological approach to globally inhibit NFAT signaling through all six zebrafish isoforms.

We first infected 2 dpf *kdrl*:*eGFP* larval zebrafish with *Mm*-tdTomato in the trunk and treated them with 125 nM FK506, a clinically utilized calcineurin inhibitor that blocks NFAT activation, for the duration of the experiment. This modest dose of FK506 was chosen due to developmental toxicities we observed at higher doses. We imaged at 4 dpi and quantitated the degree of vasculature induced in the presence and absence of inhibitor under computational blinding. Even with a low dose of FK506, we noted a small, but statistically significant, reduction in the mean degree of neovascularization at this time point, consistent with a role for NFAT in controlling angiogenesis in response to *M*. *marinum* infection (Figures 2C and 2D).^52^ To ask whether this effect was specific to recognition of TDM itself, we injected purified TDM or vehicle (incomplete Freund’s adjuvant [IFA]) alone into the trunks of 2 dpf larvae and measured the degree of angiogenesis induced. Treatment with FK506 resulted in a statistically significant reduction in the degree of angiogenesis induced at 2 dpi (Figures 2E and 2F), suggesting that this pathway was relevant specifically to TDM-mediated angiogenesis.

### The isoform NFATC2 is specifically required for mycobacteria-induced angiogenesis

Combining our observations on the correspondence of granuloma formation and the induction of *vegfaa* with our data implicating the NFAT pathway, we sought to identify NFAT isoforms that were enriched in granuloma macrophage populations. Aside from investigations made into *nfatc1*, which is restricted to the endocardium, lymphatic vessels, and the notochord during much of zebrafish development,^53–55^ little is known of the expression patterns of these genes in zebrafish, especially in the context of infection. We first made use of published scRNA-seq datasets from mycobacterial granulomas in zebrafish and non-human primates for *nfat* transcripts that were expressed in granuloma macrophages and identified both zebrafish *nfatc2a* and *nfatc3a* as plausible candidates.^13,23^

**Figure 3.**
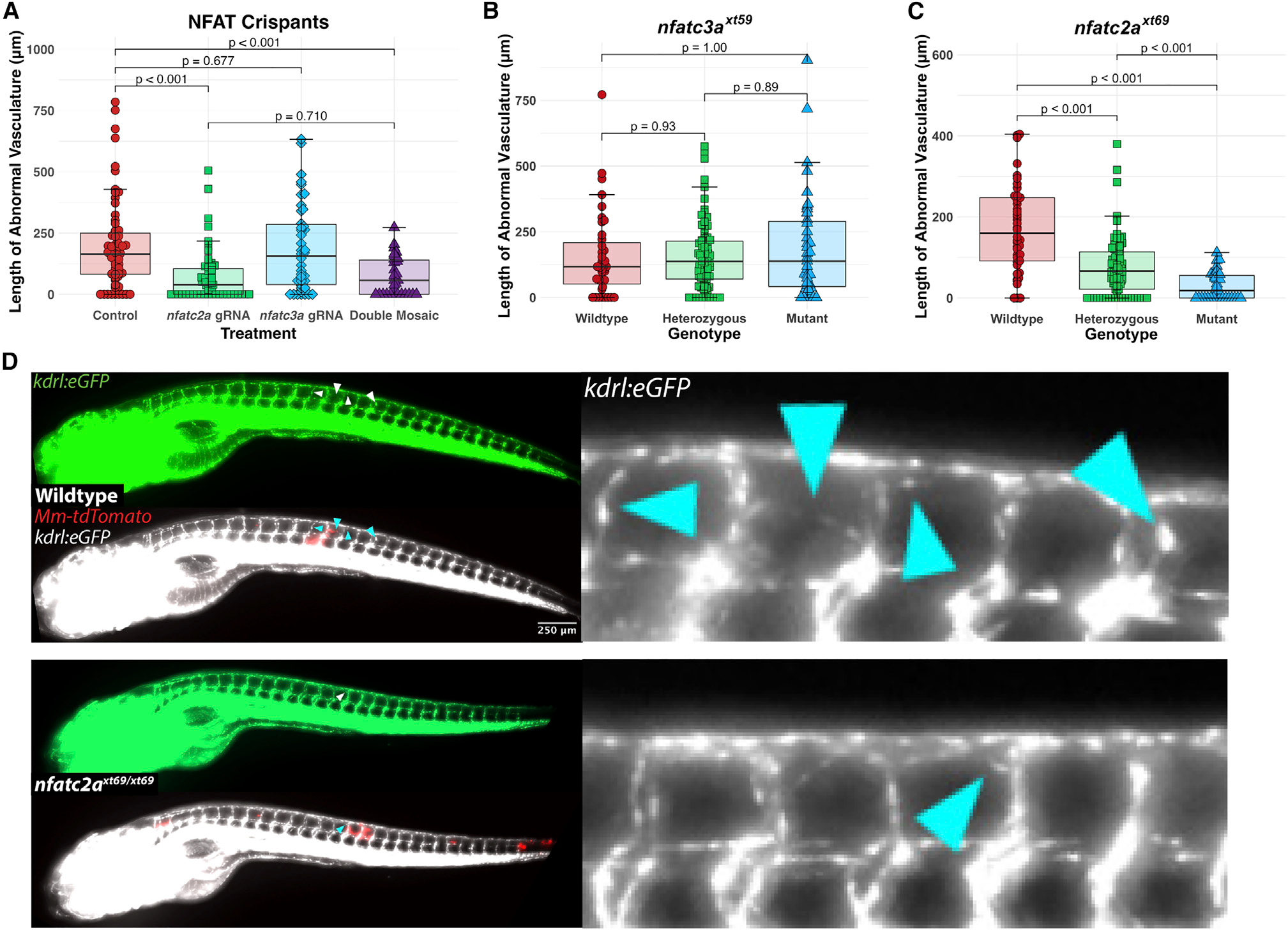
*nfatc2a*, but not *nfatc3a*, is required for the angiogenic response to mycobacterial infection *in vivo* (A) Quantitation of ectopic vasculature from sgRNP-injected, *Mm*-infected *kdrl*:*eGFP* larval zebrafish. A single high-scoring sgRNA was selected from CRISPRscan and injected either singly (*nfatc2a* gRNA, *nfatc3a* gRNA) or at an identical total RNA mass (double mosaic). At 2 dpf, these were infected along the trunk with *Mm*-tdTomato and imaged at 4 days post infection (dpi). Each point represents a single larva. Control fish are uninjected siblings. n = 63 control, 55 *nfatc2a*, 42 *nfatc3a*, 31 double mosaic. Statistics are from Dunn’s Kruskal-Wallis multiple comparisons test with Benjamini-Hochberg adjustment for independent tests. Representative of three biological replicates. Additional replicates provided in Figures S2A and S2B. (B) Blinded quantitation of angiogenic vessels from larvae from a heterozygous incross of *kdrl*:*eGFP*; *nfatc3a*^*xt59/+*^ fish demonstrated no differences across the genotypes, confirming preliminary findings in (A). Data from a single experiment, n = 39 wild-type, 89 heterozygous, 39 homozygous mutant. Statistics by Dunn’s Kruskal-Wallis multiple comparisons test with Benjamini-Hochberg adjustment. (C) Quantitation of angiogenesis from a heterozygous incross of *kdrl*:*eGFP*; *nfatc2a*^*xt69/+*^ fish under genotypic blinding. Homozygous mutant fish display an average of ~30%–90% reduction in the length of angiogenic vessels. Representative of three independent experiments (additional replicates in Figures S2E and S2F). Statistics by Dunn’s Kruskal-Wallis multiple comparisons test with Benjamini-Hochberg adjustment. n = 44 wild-type, 92 heterozygous, 35 homozygous mutant. (D) Representative images from *kdrl*:*eGFP*; *nfatc2a*^*xt69/xt69*^ and *kdrl*:*eGFP*; *nfatc2a*^*+/+*^ larvae. Vascularization in the homozygous mutant is notably reduced compared with wild type. Arrowheads indicate areas of neovascularization.

To examine potential roles for *nfatc2a* and *nfatc3a* in granuloma-associated angiogenesis *in vivo*, we first screened F_0_ CRISPR-injected mosaic knockouts (“crispants”) to rapidly evaluate these candidate genes. Using this approach, similar to that used previously by other groups, we assessed the relative roles of these two isoforms individually and in tandem, measuring the angiogenic response to mycobacterial infection in the *kdrl*:*eGFP* background under blinding.^56–59^ We found that *nfatc2a* inhibition resulted in a ~50%–80% reduction in angiogenesis at this time point. In contrast, *nfatc3a* had no effect on the length of ectopic blood vessels present. The dual-targeted double mosaics were statistically indistinguishable from the *nfatc2a*-injected fish alone (Figures 3A, S2A, and S2B). This allowed us to prospectively identify *nfatc2a* as an NFAT isoform required for full angiogenic response to mycobacteria, while *nfatc3a*, despite expression in overlapping cell populations, appeared to be entirely dispensable for this process at this time point (Figures 3A, S2A, and S2B).

We then established stable, germline transmitting indel mutant alleles for both genes to validate our results from mosaic animals. Recapitulating our results in the F_0_ generation, the *nfatc3a*^*xt59*^ mutation carrying a 22 bp deletion (leading to an early stop codon at amino acid 9 in exon 1) had no effect on angiogenesis at 4 dpi (Figures 3B and S2C). We then developed a knockout line of *nfatc2a* bearing a net 4 bp insertion leading to an early stop codon in the second exon (at amino acid 273, frameshifted after amino acid 247), prior to the DNA-binding domain (*nfatc2a*^*xt69*^) (Figure S2D). We repeated our angiogenesis assay using larvae from incrosses of *kdrl*:*eGFP*; *nfatc2a*^*xt69/+*^ animals that produced expected Mendelian ratios of wild-type, heterozygous, and homozygous mutant offspring that were assayed under genotypic blinding. Consistent with the results from mosaic animals, homozygous knockout of *nfatc2a* was sufficient to reduce the degree of angiogenesis present in larval zebrafish at 4 dpi (Figures 3C, 3D, S2E, and S2F). Importantly, given the known role of NFAT isoforms in T cell function, these defects emerged prior to the developmental emergence of functional T cells.^60^ However, whole-animal knockouts could not address potential roles for other cell types in mediating this process.

### NFAT is essential for angiogenesis induction *in vivo* in a macrophage-specific manner

**Figure 4.**
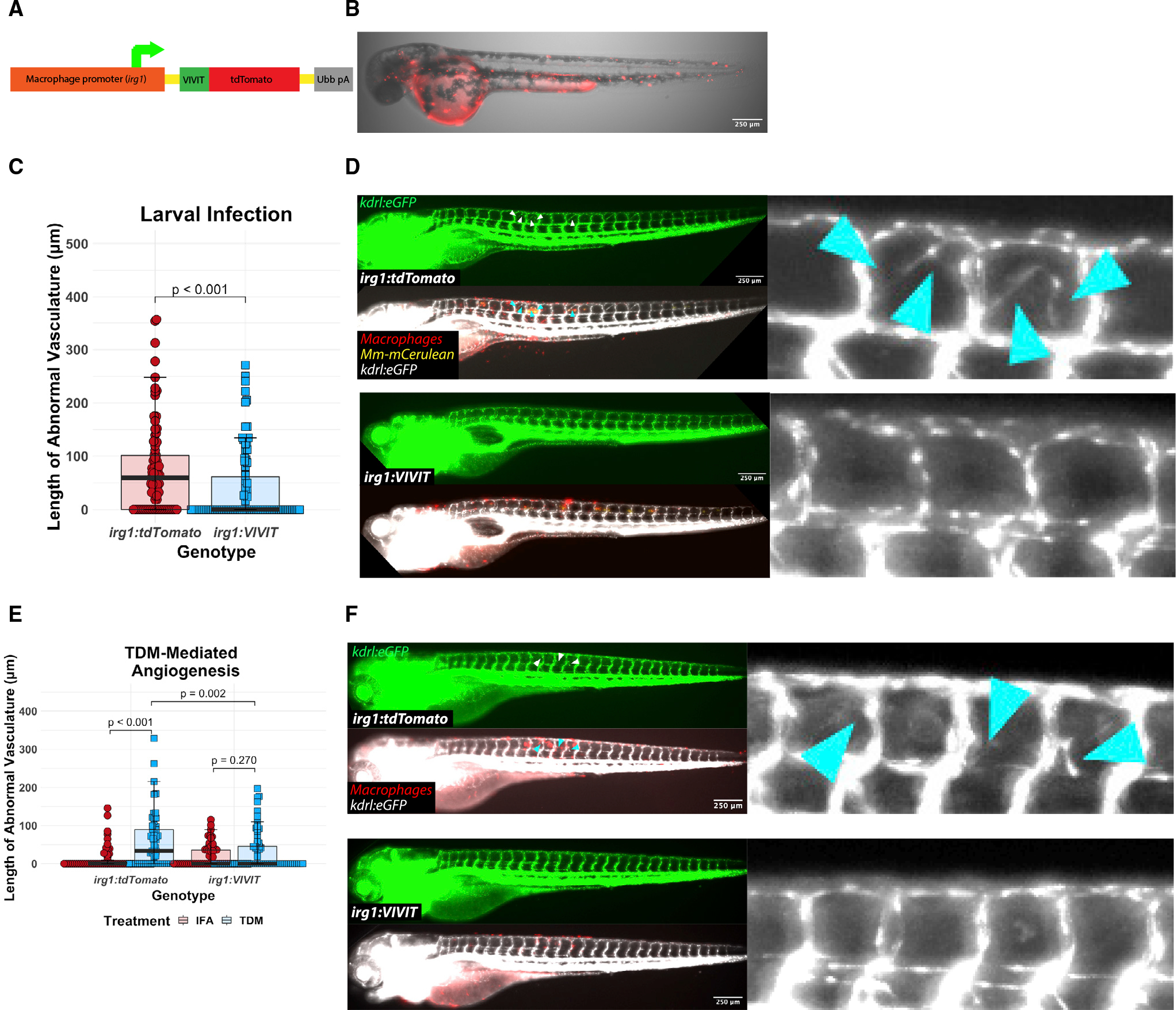
Macrophage-specific inhibition of NFAT signaling reduces angiogenesis *in vivo* during mycobacterial infection and in response to purified TDM (A) Diagram of the Tg(*irg1*:*VIVIT-tdTomato*^*xt39*^) line. The VIVIT peptide is directly conjugated to the tdTomato fluorescent protein and expression is driven by a −4.6 kb macrophage-specific *irg1* promoter. (B) Representative 2 day post fertilization larva showing macrophage-specific tdTomato^+^ expression throughout the larva. Note background expression in the yolk. Scale bar, 250 μm. (C) Quantitation of angiogenesis during *Mm* infection of *irg1*:*VIVIT-tdTomato* or *irg1*:*tdTomato* larvae. *irg1*:*VIVIT* larvae display a statistically significant reduction in the degree of angiogenesis induced by infection at 4 dpi compared with *irg1*:*tdTomato* larvae. Statistics from Wilcoxon ranked-sign test. Representative of three independent biological replicates. Additional replicates are shown in Figures S2G and S2H; n = 92 tdTomato, 98 VIVIT. (D) Representative images of *irg1*:*tdTomato* and *irg1*:*VIVIT-tdTomato* larvae at 4 dpi. VIVIT-expressing larvae display reduced neovascular elaboration compared with tdTomato-only controls. (E) Quantitation of TDM-induced angiogenesis in *irg1*:*VIVIT-tdTomato* larvae compared with *irg1*:*tdTomato* larvae. Fish were injected with either TDM emulsified in IFA or IFA alone. Statistics were conducted by Dunn’s Kruskal-Wallis multiple comparisons test with Benjamini-Hochberg adjustment. Representative of three independent biological replicates. Additional replicates provided in Figures S2I and S2J; n = 59 tdTomato/IFA, 69 tdTomato/TDM, 62 VIVIT/IFA, 71 VIVIT/TDM. (F) Representative images of TDM-injected larvae from either *irg1*:*VIVIT*-*tdTomato* or *irg1*:*tdTomato* animals. The *irg1*:*VIVIT-tdTomato* condition displays a reduction in angiogenesis to the level of background while *irg1*:*tdTomato* fish induce a robust angiogenic response. Arrowheads indicate regions of neovascularization.

Given our observations on *vegfaa* induction in macrophages at the granuloma, we tested whether NFAT signaling was required specifically in macrophages for granuloma-associated angiogenesis. For *in vivo* inhibition of macrophage NFAT signaling during infection, we developed an approach that takes advantage of the NFAT-inhibitory peptide, VIVIT, which competitively inhibits calcineurin-dependent activation of all the NFATc isoforms.^61^ This approach has been successfully used as an exogenous treatment in cell culture^35^ and mice,^51,62,63^ through ectopic overexpression in cell culture,^64^ and, more recently, in transgenic mice.^65^ We developed a transgenic zebrafish line in which VIVIT is expressed specifically in macrophages, Tg(*irg1*:*VIVIT-tdTomato*^*xt38*^) (from here, simply *irg1*:*VIVIT*) (Figures 4A and 4B).^41^ We assessed whether the macrophage-specific expression of VIVIT would be sufficient to reduce the degree of angiogenesis during infection in the trunk with wild-type *M*. *marinum* expressing mCerulean (*Mm*-mCerulean). We found that macrophage-specific VIVIT expression significantly reduced angiogenesis in response to infection (Figures 4C, S2G, and S2H). This suggested a macrophage-specific role for NFAT signaling downstream of mycobacterial detection that was necessary to induce angiogenesis, presumably through the *nfatc2a* isoform.

To ask more directly whether the decreased angiogenesis observed in the NFAT-deficient macrophages was via a TDM-mediated pathway, we used the TDM injection assay we had developed previously. We injected TDM or the IFA vehicle into the trunk of 2 dpf larval zebrafish (Figure 4D) and measured the resulting angiogenesis at 2 dpi under blinding.^25^ TDM was sufficient to induce angiogenesis *in vivo* and this effect was dependent upon functional NFAT signaling, with the degree of TDM-induced angiogenesis reduced to the level of the vehicle alone in *irg1*:*VIVIT* animals compared with *irg1*:*tdTomato* controls (Figures 4E, S2I, and S2J).

### NFAT activation is essential for angiogenesis in adult granulomas

**Figure 5.**
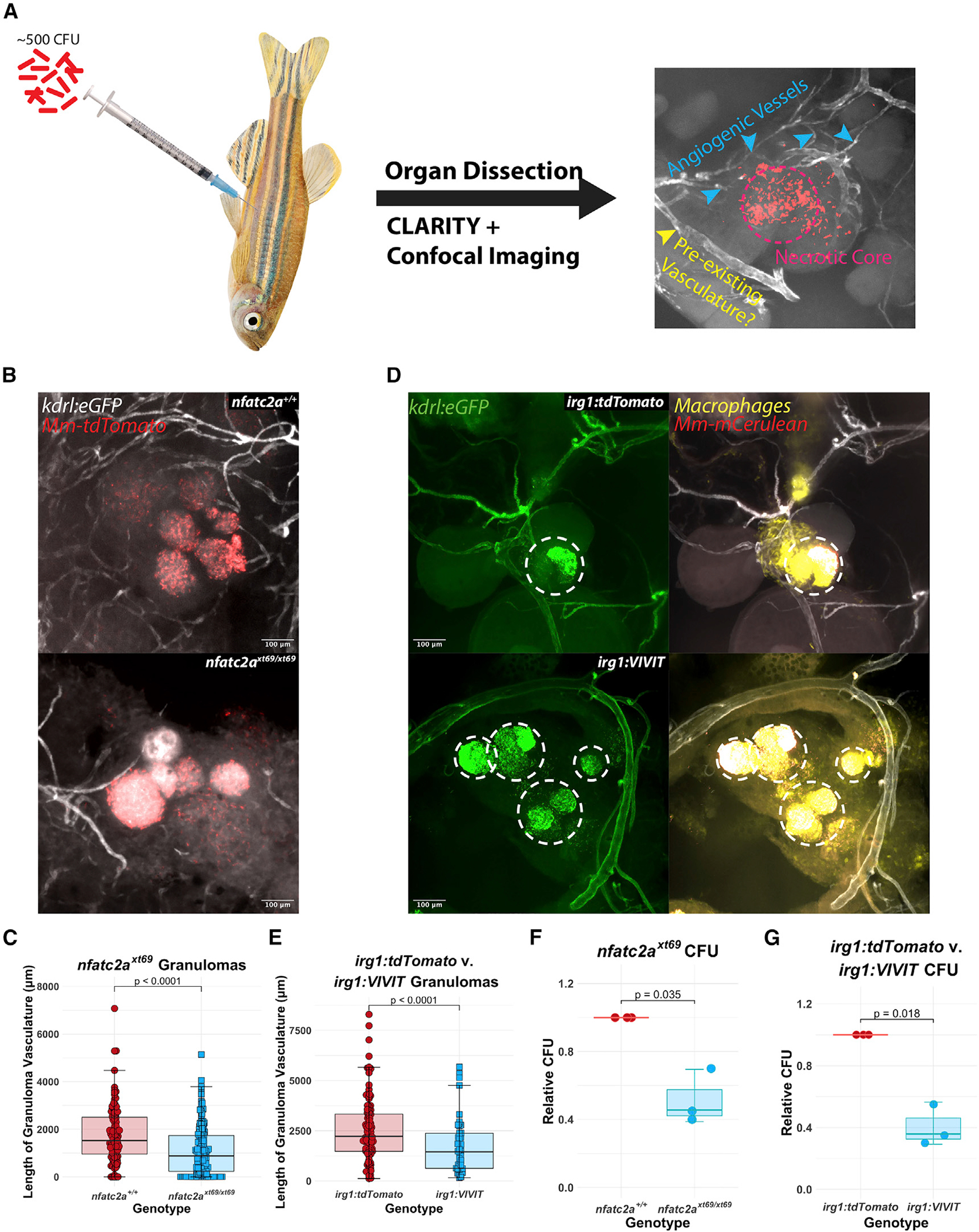
Angiogenesis in epithelioid granulomas depends on macrophage-NFAT signaling and *nfatc2a* (A) Schematic of adult infections. Adult zebrafish are infected with ~500 CFU of fluorescent mycobacteria (*Mm*-mCerulean for *irg1*:*VIVIT-tdTomato* and *irg1*:*tdTomato*, *Mm*-tdTomato for *nfatc2a*) and after granulomas have established (≥14 dpi), peritoneal organs are harvested, fixed, and cleared of birefringent lipids. After confocal imaging and Z projection, various types of vasculature can be observed, including abnormal-appearing spindles and webs of vascular sprouts as well as more-established luminal vessels that potentially comprised the existing vascular network at that location. Dashed circle delineates necrotic core; blue arrowheads neovascularization; yellow arrowheads indicate thicker vasculature, which presumably was pre-existing. (B) Representative images of *kdrl*:*eGFP*; *nfatc2a*^*xt69/xt69*^ and *kdrl*:*eGFP*; *nfatc2a*^*+/+*^ siblings. The extensive vascular network observed in wild-type animals is reduced in the *nfatc2a*^*xt69/xt69*^ animals. Note that the vessels emerge in all three dimensions around and occasionally into the outer layers of the granuloma. (C) Quantitation of the total proximal vessel length in *kdrl*:*eGFP*; *nfatc2a*^*xt69/xt69*^ and *kdrl*:*eGFP*; *nfatc2a*^*+/+*^ siblings 18 days after infection with 500 CFU. Each data point represents a single granuloma; n = 121 wild-type granulomas, 151 homozygous mutant granulomas over 6 separate animals per genotype. Representative of 3 independent experiments. Quantitation was performed under programmatic blinding. *nfatc2a*^*xt69/xt69*^ fish display ~40%–70% reduction in average sum vascular network length compared with wild-type siblings (see also Figures S3A and S3B). Statistics from Student’s t test. (D) Representative images of *irg1*:*VIVIT-tdTomato*; *kdrl*:*eGFP* and *irg1*:*tdTomato*; *kdrl*:*eGFP* granulomas. Dashed circles highlight the necrotic cores, which, due to both autofluorescence and leaky emission capture from the *Mm*-mCerulean bacteria, bleed into the GFP channel. *irg1*:*tdTomato* fish display robust interpenetration of blood vessels into and around the granuloma while little angiogenesis is seen in the *irg1*:*VIVIT-tdTomato* condition (see also Figure S3C). Note the large, luminal vessel in the *irg1*:*VIVIT-tdTomato* granuloma, which appears mature and may have existed preceding infection, but is included in quantitation. (E) Quantitation of the total vessel length in *irg1*:*VIVIT-tdTomato*; *kdrl*:*eGFP* and *irg1*:*tdTomato*; *kdrl*:*eGFP* granulomas. Each data point represents a single granuloma; n = 84 tdTomato, 74 VIVIT granulomas over 6 independent animals per genotype. Representative of three independent experiments. Quantitation was performed under programmatic blinding. *irg1*:*VIVIT-tdTomato* fish display a 30%–60% reduction in total vessel length compared with *irg1*:*tdTomato* (see also Figures S3D and S3E). Statistics are from Student’s t test. (F) Enumeration of bacterial burden from whole infected adult zebrafish 24 dpi. *nfatc2a*^*xt69/xt69*^ and *nfatc2a*^*+/+*^ siblings were infected with 1,000 CFU *Mm*-tdTomato. The experiment was performed independently three times and each data point represents the median CFU from up to eight fish within a single experiment normalized to the wild-type siblings for that replicate. *nfatc2a*^*xt69/xt69*^ fish had a ~55% median reduction in CFU compared with wild type. Statistics from Student’s t test comparing median CFU values from each of three independent experiments, each with eight fish per genotype. (G) Enumeration of bacterial burden in whole infected adult zebrafish 18 dpi. *irg1*:*VIVIT-tdTomato* and *irg1*:*tdTomato* fish were infected with 1,000 CFU *Mm*-tdTomato. Each data point is the median CFU value from an independent experiment and each experiment contains up to eight fish of each genotype. *irg1*:*VIVIT-tdTomato* fish had a ~65% median reduction in CFU compared with control fish. Statistics from Student’s t test comparing median CFU values from each of three independent experiments, each with eight fish per genotype.

Adult zebrafish are equipped with both innate and adaptive immunity and form mycobacterial granulomas that histologically mirror epithelioid human tuberculosis granulomas,^16^ including induction of a surrounding vascular network. To assess whether our findings in the larvae translated to longer-term infections in the presence of adaptive immunity, we infected adult *kdrl*:*eGFP*; *nfatc2a*^*xt69/xt69*^ zebrafish and *kdrl*:*eGFP*; *nfatc2a*^*+/+*^ siblings with *Mm*-tdTomato and examined organs at 18 dpi after CLARITY-based clearing.^66,67^ Cleared organs were then imaged by spinning disk confocal microscopy (Figure 5A). We measured the total vascular network surrounding the granulomas in a programmatically blinded fashion^68^ and found that *nfatc2a*^*xt69/xt69*^ fish had a significant reduction (~50%) in the length of the vascular network compared with wild-type siblings, further validating this gene as important for the angiogenic response *in vivo* (Figures 5B, 5C, S3A, and S3B). These putatively neovascular vessels tend to be highly branched and to be comprised of a limited number of cells with small or non-existent luminal volume, indicating that they are still in the sprouting stage of angiogenesis and suggesting a potential failure to mature. We observed robust effects that are likely understated in our quantitation, as we could not formally make any distinction between thicker, pre-existing vasculature that happens to lie nearby the site of granuloma formation and the characteristic neovascularization more intimately associated with the granuloma that is present in wild-type animals but reduced in *nfatc2a* mutants (Figures 5A and S3C).

### Macrophage-specific NFAT inhibition in mature granulomas reduces angiogenesis

We next evaluated whether macrophage-specific NFAT inhibition had similar effects on vascularization in adult zebrafish. We infected adult *irg1*:*VIVIT*; *kdrl*:*eGFP* and *irg1*:*tdTomato*; *kdrl*:*eGFP* double transgenic zebrafish with *Mm-mCerulean* and examined visceral organs at 14 dpi. We used confocal imaging to visualize individual CLARITY-cleared organs and measured the total length of granuloma-proximal vasculature under blinding as above.^68^ We found that the degree of vascularization was significantly reduced around granulomas from *irg1*:*VIVIT* fish compared with *irg1*:*tdTomato* fish (Figures 5D, 5E, S3D, and S3E). The extent of the vascular network in the *irg1*:*VIVIT* condition was notably restricted or solely comprised of more mature, luminal vessels, suggesting a total failure to induce an angiogenic response (Figure 5D). These findings, consistent with our previous data from both larval zebrafish infections in the *irg1*:*VIVIT* background and in the *nfatc2a* mutant adult fish, point to a critical role for macrophage-specific NFAT activation in inducing the angiogenic response at mycobacterial granulomas. Furthermore, this establishes that NFAT function is broadly conserved from early larval infection through to the mature necrotic granulomas that characterize adult infection.

### Inhibition of NFAT signaling results in decreased bacterial burden

We had previously shown that inhibition of granuloma-associated vascularization is associated with decreased bacterial burden. Mycobacterial mutants unable to induce vascularization (Δ*pcaA*), as well as either genetic or pharmacological inhibition of VEGF signaling all result in reduced bacterial burden, presumably due to functions of the aberrant vasculature promoting bacterial growth and/or inhibiting bacterial killing.^18,24,25^ To examine the effect on burden of inhibition of NFAT signaling, we performed colony-forming unit (CFU) assays at time points after the induction of angiogenesis and granuloma maturation. We infected *nfatc2a*^*+/+*^ and *nfatc2a*^*xt69/xt69*^ adult zebrafish with *Mm*-tdTomato and plated them for CFU at 24 dpi. We found that knockout of *nfatc2a* resulted in a ~50% decrease in median colony number compared with wild-type animals after extended infection (Figure 5F).

Finally, we evaluated the impact of macrophage-specific NFAT inhibition on whole organism bacterial burden. We infected adult zebrafish possessing either the *irg1*:*VIVIT* or *irg1*:*tdTomato* transgenes with *Mm*-tdTomato and then homogenized and plated these fish at 18 dpi. We found that macrophage expression of the VIVIT peptide resulted in a median reduction of ~60% of the bacterial burden in these fish at this time point relatively soon after the formation of necrotic granulomas and robust induction of angiogenesis (Figure 5G).

### Pharmacological inhibition of NFAT in human THP-1 macrophages limits VEGFA induction by *Mtb*

**Figure 6.**
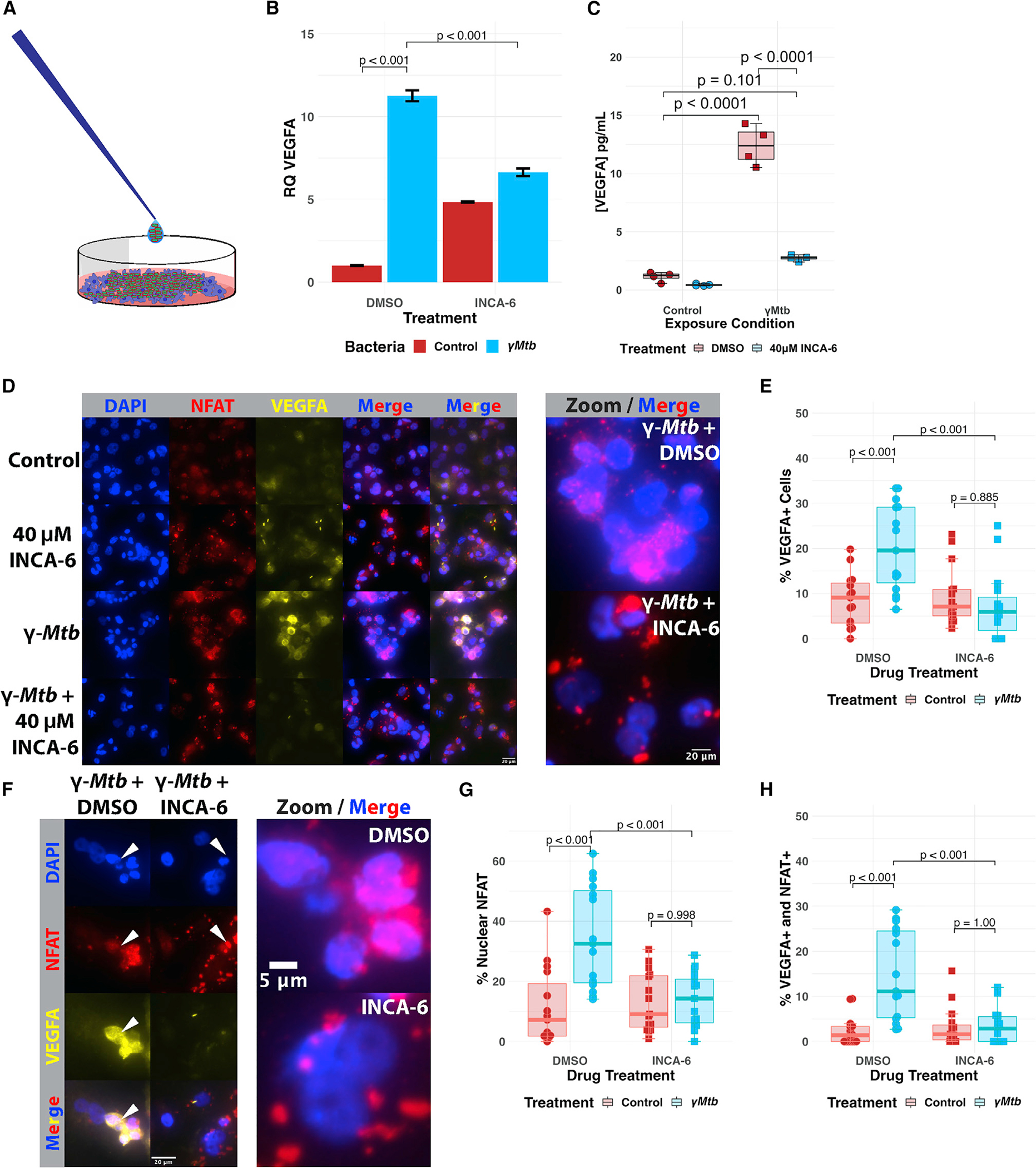
Pharmacological inhibition of NFAT in THP-1 human macrophages restricts *VEGFA* transcriptional induction and VEGFA production in response to *Mtb* (A) Pictorial representation of the γ-irradiated *Mycobacterium tuberculosis* (γ*Mtb*) exposure assay. Partially sonicated clumps of γ*Mtb* are overlaid on THP-1 monolayers to model a primarily extracellular route of exposure that might be seen during longer courses of infection, such as in the granuloma. (B) qRT-PCR analysis of γ*Mtb*-exposed THP-1 macrophages at 8 h post exposure. *VEGFA* transcripts are upregulated in response to γ*Mtb*/DMSO-treated cells, but this upregulation is suppressed by treatment with 40 μM INCA-6, a chemical inhibitor that interferes with calcineurin binding to NFAT. Each data point represents the mean relative quantity from three technical replicates for each biological replicate within the single experiment; representative of three independent experimental replicates. Additional replicates are provided in Figures S4A and S4B. Statistics from ANOVA with Tukey post-hoc honest significant differences test. (C) Supernatant ELISA of THP-1 macrophages at 24 h post exposure reveals potent induction of VEGFA only in the γ*Mtb*/DMSO-treated group. Representative of three independent experiments. Statistics from ANOVA with Tukey post-hoc honest significant differences test. (D) Immunofluorescence imaging reveals upregulation of VEGFA in γ*Mtb*-exposed THP-1 cells and substantial NFATC2 upregulation and nuclear translocation after treatment. Effects are both inhibited by treatment with INCA-6. DAPI channel labels nuclei; NFAT is detected by staining with anti-NFATC2 antibody; VEGFA detected by staining with anti-VEGFA antibody. Representative of three independent biological replicates. (E) Quantitation of the percentage of VEGFA^+^ cells within entire images for (D). The first five images from each experimental group were selected and computationally blinded. Total cell number was counted in addition to the number of VEGFA^+^ cells in each field. Each data point represents the percentage of VEGFA^+^ cells in the field; 15 (5 of each of 3 total replicates) total fields were counted for each groups (5 from each of 3 independent experiments). Total number of cells counted = 5,264. Statistics from ANOVA with Tukey honest significant differences test. (F) Magnified immunofluorescence images demonstrating VEGFA inhibition by addition of INCA-6 with γ*Mtb*-exposed THP-1 cells. Note the white arrows showing nuclear localization of NFATC2 corresponding with VEGFA signal in the DMSO-treated condition while NFATC2 nuclear localization is disrupted in the INCA-6 condition and little VEGFA can be observed. (G) Quantitation of the percentage of cells with nuclear NFAT residence from the same images as (F). Each data point represents the percentage of NFAT nuclear-resident cells in the field; 15 total fields were counted for each groups (5 from each of 3 independent experiments). Total number of cells counted = 5,264. Statistics from ANOVA with Tukey honest significant differences test. (H) Quantitation from (F and G) of the percentage of total cells with nuclear NFAT residence and also expressing VEGFA out of the total number of cells. Each point represents one of 15 total fields counted for each group (5 from each of 3 independent experiments). Total number of cells counted = 5,264. Statistics from ANOVA with Tukey honest significant differences test.

The zebrafish mycobacterial infection model shares important conserved features with *Mtb* infection of humans, host response, and granuloma angiogenesis.^13,16,18,20^ In addition, important aspects of the response to cyclopropanated TDM appear to be largely maintained between zebrafish and humans.^25^ We next asked whether our findings discovered *in vivo* with the zebrafish-*M*. *marinum* model were conserved in human cells exposed to *Mtb*. We developed a cell culture model of macrophage-*Mtb* interactions using differentiated THP-1 monocytic cells exposed extracellularly to γ-irradiated *Mycobacterium tuberculosis* H37Rv (γ*Mtb*), which produces the full spectrum of TDM species, presented to the cell in their native configuration (as compared with heat-killed *Mtb*, which disrupts cell envelope structure and organization)^69,70^ (Figure 6A). We found that exposure of differentiated THP-1 macrophages to γ*Mtb* was sufficient to induce *VEGFA* transcription as well as VEGFA secretion (Figures 6B and 6C). To examine whether NFAT signaling is required for production and secretion of VEGFA, we treated THP-1 macrophages with the small-molecule inhibitor INCA-6, which specifically disrupts the interaction between the NFAT family members and their activating phosphatase, calcineurin.^71^ Strikingly, treatment of THP1 cells with INCA-6 during γ*Mtb* exposure significantly inhibited transcriptional induction of *VEGFA* to levels near drug treatment alone (Figures 6B, S4A, and S4B), as well as VEGFA secretion (Figures 6C, S4C, and S4D). Immunofluorescence revealed robust translocation of NFAT (using an NFATC2 antibody) that was broadly correlated to VEGFA signal (Figures 6D and 6F). These effects were then quantified in a blinded fashion, demonstrating robust VEGFA expression in γ*Mtb*-exposed cells that is inhibited by INCA-6 (Figure 6E). Similarly, γ*Mtb* induced robust NFAT nuclear localization that was able to be inhibited by INCA-6 (Figure 6G). We then investigated the overlap between these two phenotypes and found that the percentage of cells expressing both VEGFA and displaying nuclear NFAT expression was increased after γ*Mtb* treatment, demonstrating a correlation between NFAT nuclear localization and VEGFA expression after macrophage detection of *Mtb* (Figure 6H). These inhibitor studies suggest that human NFAT signaling is required for VEGFA production in response to *Mtb* exposure.

### Role of human NFATC2 in VEGFA induction

**Figure 7.**
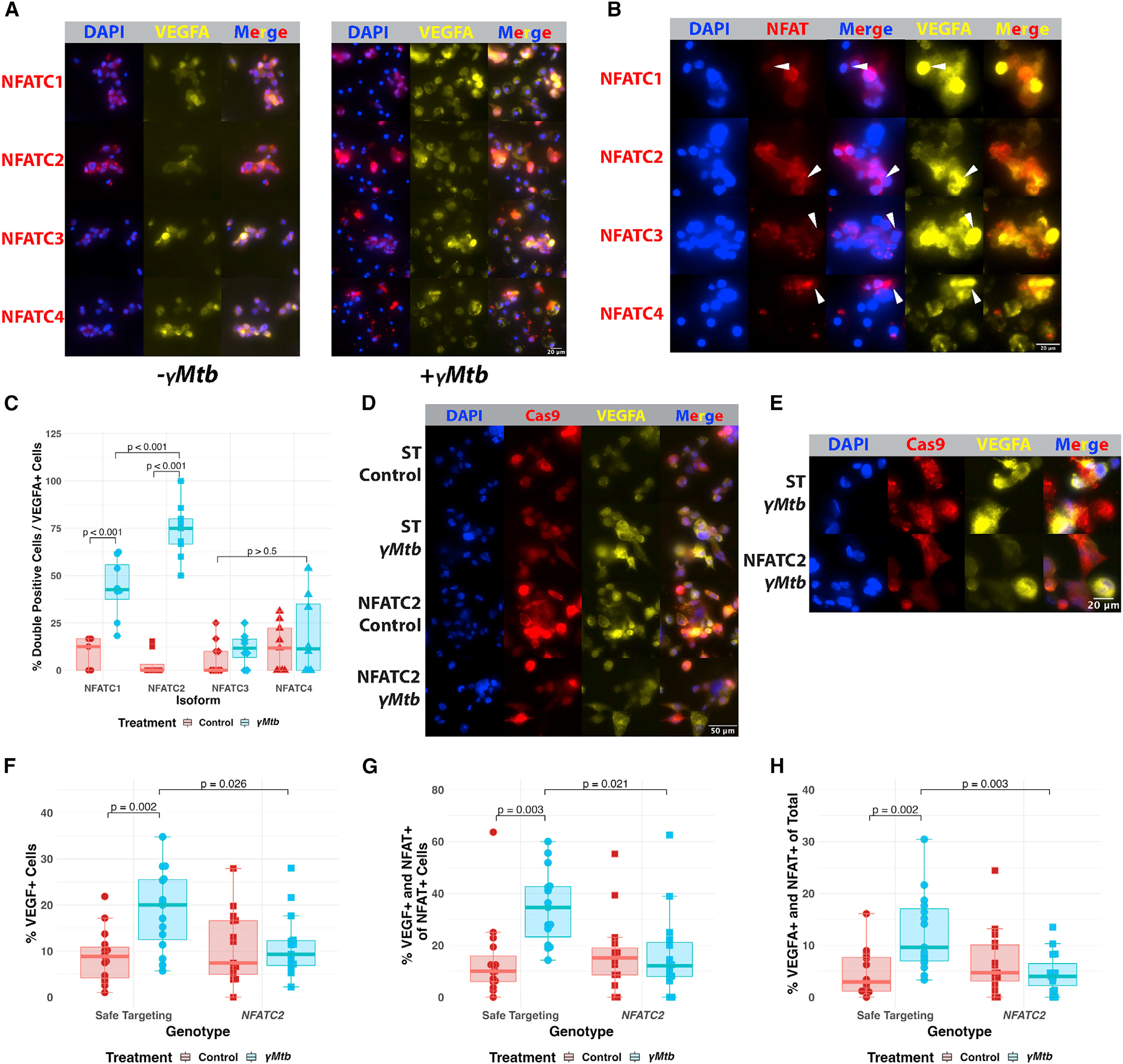
*NFATC2* is required for the VEGFA response of cultured macrophages to γ*Mtb* exposure (A) Immunofluorescence staining of THP-1 macrophages in the presence or absence of γ*Mtb* to identify potential NFAT isoforms of interest in human cells. NFATC2 is most robustly expressed and inducibly nuclear of these isoforms in macrophages. NFATC1 and NFATC3 are comparatively less expressed and less extensively translocated by 8 h post exposure. NFATC4 is very lowly expressed initially but, like all the isoforms, appears to be upregulated at the protein level after γ*Mtb* exposure. Initial staining from a single experiment, with NFATC2 validation from three biological replicates in (D–G). (B) Magnified images showing robust VEGFA expression in cells with nuclear NFATC2 compared with cells with nuclear NFATC1, NFATC3, and NFATC4, which lack the strong correspondence between NFAT nuclear localization and VEGFA induction, which is only seen with NFATC2 staining. (C) Blinded quantitation of the relationship between each NFAT isoform and the induction of VEGFA. We calculated the subset of cells expressing both VEGFA and demonstrating obvious NFAT nuclear localization and normalized to the total number of cells expressing VEGFA in that field. Initial staining from a single experiment, with validation in (D–G). Total number of cells counted = 5,859. Statistics from ANOVA with Tukey honest significant differences test. (D) THP-1 cells transduced with Cas9-expressing lentiviruses targeting either NFATC2 (Figures S4F and S4G) or safe-targeting loci (Figure S4H) were selected with puromycin and then treated with γ*Mtb* or vehicle. Safe-targeting-transduced THP-1 cells robustly responded to γ*Mtb* with VEGFA production at 8 h post exposure, but fewer NFATC2-transduced cells produce VEGFA after stimulation and at lower staining intensity. Representative of three biological replicates. (E) Magnified images showing high VEGFA induction in γ*Mtb*-treated NFATC2-targeted THP-1 cells compared with γ*Mtb*-treated safe targeting (ST) THP-1 cells. The ST cells show robust VEGFA induction and NFAT nuclear translocation while NFATC2 cells show diminished VEGFA induction and disordered nuclear translocation and occasional lack of NFATC2 staining entirely; the antibody used to detect NFATC2 is N-terminal to the sgRNA sites, so residual expression is likely captured by the antibody. Representative of three biological replicates. (F) Blinded quantitation of the percentage of VEGFA^+^ cells within entire images for (D and E). Each data point represents the percentage of VEGFA^+^ cells in the field; 15 total fields were counted for each groups (5 from each of 3 independent experiments). Total number of cells counted = 5,029. Statistics from ANOVA with Tukey honest significant differences test. (G) Blinded quantitation of the percentage of VEGFA^+^ and nuclear-localized NFAT out of the total number of cells with NFAT nuclear localization within entire images for (D) and (E). Fifteen total fields were counted for each groups (5 from each of 3 independent experiments). Total number of cells counted = 5,029. Statistics from ANOVA with Tukey honest significant differences test. (H) Blinded quantitation of the percentage of VEGFA^+^ and nuclear-localized NFAT of the total number of cells in the images for (D) and (E). Fifteen total fields were counted for each group (5 from each of 3 independent experiments). Total number of cells counted = 5,029. Statistics from ANOVA with Tukey honest significant differences test.

To identify functionally important NFAT human isoforms, we exposed THP-1 macrophages to γ*Mtb* and examined expression and localization of specific NFAT isoforms and their relationship to VEGFA expression. To visualize VEGFA expression in individual cells, we used the secretion inhibitor brefeldin A to lock VEGFA within secreting cells. Simultaneous staining for each of the four human NFATc proteins along with VEGFA allowed us to identify NFAT isoforms that underwent changes in expression and localization and correlate this with VEGFA production (Figure 7A). While THP-1 macrophages express all of the isoforms to varying degrees, the most intense co-staining with VEGFA was found with NFATC2 (Figure 7B). In addition, while each of the isoforms showed alterations after γ*Mtb* exposure, only NFATC2 (and to a lesser extent, NFATC1) showed robust nuclear localization that appeared to correspond to VEGFA induction in individual cells (Figures 7C and S4E). While NFAT isoform translocation was observable with NFATC1, this correspondence was notably weaker than that with NFATC2. Given the strong correlation for NFATC2 with nuclear localization and VEGFA production after γ*Mtb* exposure, expression data from zebrafish and non-human primate granulomas, as well as the *in vivo* zebrafish results implicating macrophage Nfatc2a in *Vegfaa* production and angiogenesis, we focused on human NFATC2 as a key isoform.

To test a functional role for human NFATC2 in macrophage induction of VEGFA during γ*Mtb* exposure, we used a lentivirus-mediated CRISPR-Cas9 approach based on approaches used in the zebrafish^59^ to introduce high-efficiency disruption of *NFATC2*. We compared these cells with those transduced with lentiviruses expressing safe-targeting control sgRNAs. (Figures S4F–S4I).^72–75^ We simultaneously expressed four distinct guide RNAs targeting *NFATC2* or safe-targeting controls, to maximize the percentage of puromycin-resistant cells possessing complete null mutations.^59^ Due to technical challenges associated with long-term culture of THP-1 cells and to address heterogeneity among cellular responses, we focused these assays on VEGFA induction in these cells by immunofluorescence after γ*Mtb* exposure. Because the N-terminal epitope recognized by our NFATC2 antibody was upstream of the targeted sites, we were unable to examine functional protein levels directly and simultaneously in the immunofluorescence images (Figure S4H), but we did observe transcriptional knockdown of *NFATC2* by qRT-PCR (Figure S4J). However, we found that transduced cells targeted by *NFATC2* lentivirus generally failed to induce VEGFA in response to γ*Mtb* exposure, while safe-targeting control lentivirus-transduced cells responded normally (Figures 7D and 7E). Thus, macrophage NFATC2-mediated induction of VEGFA downstream of mycobacterial TDM exposure is conserved from zebrafish to human cells exposed to *M*. *tuberculosis*. We quantified these effects in a blinded manner and found that, indeed, NFATC2-targeted THP-1 macrophages failed to induce VEGFA (Figure 7F) and failed to induce NFATC2 in a manner that correlated with VEGFA expression, suggesting functional disruption of the protein (Figure 7G). We lastly measured the total number of VEGFA-positive cells with NFATC2 nuclear localization and found a significant decrease after *NFATC2* targeting (Figure 7H), suggesting functional disruption of the association between these phenomena.

## DISCUSSION

This work uncovers an unexpected role for macrophage NFAT activation in immune responses to pathogenic mycobacteria and the maladaptive angiogenic responses that occur during infection. Activation of NFAT is driven through recognition of bacterial cyclopropanated TDM, a major constituent of the cell envelope in pathogenic mycobacteria, which we have previously found is necessary and sufficient to drive pathological angiogenesis.^25^ Identifying this unanticipated role for NFAT in angiogenesis expands our understanding of the mechanisms governing mycobacterial pathogenesis and offers targets for potential host-directed therapeutics. Traditionally, work on TDM-mediated C-type lectin activation has focused on CARD9 and NF-κB signaling. Here, in contrast, we describe a specific role for alternative C-type lectin signaling responses through the NFAT pathway to drive VEGFA production and granuloma-associated angiogenesis.

VEGFA induction is a prominent feature of TB in human disease as well as in a number of animal models, including non-human primates, rabbits, mice, and zebrafish.^13,18,20,23,19^ We found that VEGFA was produced specifically within newly arrived macrophages at nascent granulomas. Macrophage populations are critical to VEGFA induction during mycobacterial infection,^18^ and macrophage-specific inhibition of NFAT signaling as well as deletion of *nfatc2a* result in reductions in granuloma-associated angiogenesis. Using a human cell culture model, we found that NFATC2 was similarly engaged in human cells as among all NFAT isoforms, NFATC2 underwent the most robust nuclear translocation in response to *M*. *tuberculosis* stimulation, which strongly correlated with VEGFA production (Figure 7C). Correspondingly, pharmacological inhibition of NFAT signaling in human cell culture as well as genetic inhibition of NFATC2 resulted in reduced VEGFA production.

Although animal models of and human patients with TB generally report high VEGFA levels, there are few studies that center on VEGFA induction in cell culture infection models.^21^ Through high-resolution time lapses and reporter lines, we found that *vegfaa* induction generally does not occur until the formation of initial granulomas and is generally correlated with the appearance of extracellular bacteria that could be recognized by incoming, likely uninfected macrophages. This concentration-dependent effect on signaling may reflect key aspects of the disease itself, wherein large numbers of extracellular bacteria can accumulate in the necrotic core of the granuloma, potentially triggering relatively insensitive and/or chronic C-type lectin signaling in this context.

Consistent with the recognition of extracellular bacteria, exposure of human macrophage-like cells to γ-irradiated *M*. *tuberculosis* rapidly induced VEGFA in an NFATC2- and dose-dependent manner. Standard cell culture infection models generally eliminate extracellular bacteria using gentamicin treatment and media changes, and so it is possible that engagement of this pathway by extracellular bacteria or TDM stimulation is a key component of this response. A survey of the literature and a variety^76–80^ of RNA-seq datasets from macrophage-*Mtb* infection experiments reveal modest or nonexistent induction of *VEGFA*, further supporting the notion that extracellular exposure to *Mtb* may be an important element of the angiogenic response and may reflect some aspects of the macrophage-*Mtb* interface within granulomas.

As its name suggests, the NFAT pathway plays an indispensable role in normal T cell biology. Accordingly, whole animal knockouts of NFAT in standard mouse models of *M*. *tuberculosis* infection—where granuloma formation itself may be limited—may have obscured a role for myeloid-specific effects of NFAT signaling.^81^ The zebrafish model, by looking at early time points, uncovered a role both in angiogenesis and, presumably as a consequence, bacterial control. Wholesale, longer-term inactivation of NFATC2, which also plays important roles in T cells, might compromise important aspects of a productive adaptive immune response during mycobacterial infection, and our adult infection studies were over relatively short time frames (2–3.5 weeks). Murine studies, indeed, show increased susceptibility to *M*. *tuberculosis* in knockouts via compromised production of IFN-γ by CD4^+^ T cells.^81^ While genetically manipulable animal models allow for cell-specific separation, any host-based therapeutic approaches might require cell-specific macrophage delivery methods,^82–84^ NFATC2-specific targeting,^72^ and/or contending with the adaptive immune response, an important aspect of host resistance during mycobacterial infection. In addition, the potential influence of NFATC2 and VEGFA on non-angiogenic pathways implicated in granulomatous inflammation will be of further interest and may impact any targeting approaches.^19^

It remains unclear why NFATC2, but not any of the other isoforms, is specifically required in macrophages for the induction of *VEGFA*, given evidence that the others are present in resting macrophages (Figure 7A). The functional distinctions between the isoforms have long been of basic interest, but relatively few specific differences between them have been identified beyond basal regulation to provide tissue specificity and more recent findings describing layers of kinetic regulation with isoform-specific stimulation thresholds, nuclear retention, and more.^85–89^ It is intriguing that this literature has implicated NFATC2 in particular in a slower and more sustained process of activation relative to the other *NFATc* paralogs.^88,89^ It will be interesting to examine potential roles for calcium dynamics as well as any potential alternative regulatory mechanisms of NFATc expression or activation during infection. These levels of regulation offer opportunities for uncovering features of the cell biology of NFAT.

Here, we identify the unique requirement for this single isoform in macrophages to induce angiogenesis in response to mycobacterial infection. One hypothesis is that NFATC2 has binding partner(s) unique among NFAT isoforms required for its effect on the *VEGFA* promoter. Whether this is HIF-1α (the canonical regulator of *VEGFA*) or one of the many previously described interacting partners is, as yet, unknown, but could be tested either *in vitro* or *in vivo* with genetic or chemical approaches. However, higher-order regulatory mechanisms that result in the production of VEGFA in the absence of overt hypoxia have been understudied and this work proposes at least one potentially generalizable mechanism whereby NFATC2 activation results in *VEGFA* transcriptional upregulation, a process that can be inhibited with chemical and genetic intervention. Despite the widespread presence of putative NFAT binding motifs (5′-GGAAA-3′) (Figure S4K) in the proximal *VEGFA* promoter,^90^ their influence on *VEGFA* transcription has been relatively unexplored as this specific effect is generally not seen in T cells or other cell types.^91^ NFAT involvement in the induction of a variety of cytokines is well documented, but which, if any, are at play in the macrophage-*Mtb* interaction is a promising subject for future research.

A more comprehensive characterization of NFAT-dependent innate immune responses has begun in recent years,^35,65,92^ but this pathway has remained unstudied in the context of macrophage signaling during mycobacterial infection. Furthermore, this work draws a connection between the induction of calcium fluctuations—which can occur in response to many different developmental, homeostatic, and pathological stimuli, including to mycobacterial infection^93–95^—to the angiogenic response to that stimulation. Our identification of NFAT regulation of VEGFA offers an approach to both pro- and anti-angiogenic intervention in various pathological contexts.

### Limitations of the study

While we have identified interesting macrophage biology mediating an important host immune response during mycobacterial infection, there are no data as to whether this might translate to other disease contexts, especially those with a prominent role for C-type lectin signaling. Whether or not this mechanism is broadly generalizable is important to understanding key aspects of pro-angiogenic macrophage behavior. In addition, we have validated important aspects of our observations in the zebrafish with a mammalian cell culture model, but subsequent studies may warrant further integration of mammalian models of TB where angiogenesis is present or human patient samples to better understand additional aspects of this process.

## STAR⋆METHODS

### RESOURCE AVAILABILITY

#### Lead contact

Further information and requests for resources should be directed to and will be fulfilled by the lead contact, David Tobin (david.tobin@duke.edu).

#### Materials availability

All materials and lines generated in this study are available from the lead contact. All component plasmids have been deposited to Addgene and are available using the stock numbers listed in the STAR Methods; final constructs are also available upon request but we recommend groups construct expression clones from the available entry and destination vectors, due to potential for recombination in the final constructs with repeated recloning and outgrowth. All other reagents are also available upon request.

#### Data and code availability

- Data availability: All raw or minimally processed images and raw image and other quantitation data (as .csv files) are publicly archived via Zenodo: https://doi.org/10.5281/zenodo.6816429. Any raw images unable to fit within the file size limits are available upon request.
- Code availability: All original code, including R and Python scripts used for processing and analysis are publicly available as of the date of this publication via Zenodo: https://doi.org/10.5281/zenodo.6816429.
- Any additional information required to reanalyze the data reported in this paper is available from the lead contact upon request.

All raw image quantitation data (as .csv files) along with R scripts used for analysis are publicly archived via Zenodo: https://doi.org/10.5281/zenodo.6816429. Where appropriate and feasible, minimally processed image files have been included. Raw image files are available upon request due to their large file size. Additionally, any ImageJ macros (in .py format) used for analysis are included for user convenience.

### EXPERIMENTAL MODEL AND SUBJECT DETAILS

#### Zebrafish husbandry

All zebrafish husbandry and experimental procedures were performed in accordance and compliance with policies approved by the Duke University Institutional Animal Care and Use Committee (protocol A091-20-04). Adult zebrafish are housed in a continuously recirculating system maintained at 28.5°C on a 14hr–10hr light-dark cycle and kept in either 3 or 6 L tanks. Reverse osmosis water was maintained at 600–700 μS conductivity by addition of Instant Ocean Sea Salt (#SS15-10) and a pH between 7.0 and 7.4 (buffered by automated addition of sodium bicarbonate; Arm & Hammer Pure Baking Soda [#426292]).

Larval zebrafish were euthanized prior to 8 days post fertilization (dpf). Sex is indeterminate at this stage in zebrafish and no distinctions are made between putatively future male or female larvae in this study. Larvae are maintained at 28.5°C in 100 mm petri dishes (Sarstedt #83.3902.500) in 50 mL of sterile E3 medium (5 mM NaCl (Fisher Scientific #S271), 178 μM KCl (VWR #BDH9258), 328 μM CaCl_2_ (VWR #BDH9224), 400 μM MgCl_2_ (Ward’s Scientific #470301)) at no more than 150 larvae per dish. For imaging, 1 dpf larvae are transferred to E3 supplemented with 1-phenyl-2-thiourea (PTU, Sigma-Aldrich #P7629) at a final concentration of 45 μg/mL to prevent melanization.

Infected adult zebrafish are kept on an identical 14hr–10hr light cycle at 28.5°C in an isolated incubator (ThermoFisher #PR505755L). Approximately equal numbers of each sex are used in experiments. Fish are kept at no greater than 1 fish/100 mL of water in crossing cages (Aquaneering #ZHCT100) with daily food (Skretting #GEMMA Micro 500) and water changes using system fish water. Fish are euthanized when showing overt signs of distress (inability to right, flared scales, labored breathing) or at the terminal time point of the experiment.

To minimize distress, anesthesia was performed in all cases prior to manipulation of both larval and adult zebrafish by the addition of MS-222 (Tricaine-S, Syndel #ANADA 200–226) at a final concentration of approximately 160 μg/mL. Duration of anesthesia was minimized to the time required to complete the manipulation or imaging.

#### Mycobacterium marinum

All strains are derived from *M*. *marinum* strain M (ATCC #BAA-535).^118^ Hygromycin-resistant fluorescent strains expressing the tdTomato,^18^ mCerulean, or EBFP2 fluorescent proteins have been described previously.^44^ Bacterial culture was carried out on either 7H10 agar (Difco #262710) plates supplemented with Middlebrook OADC growth supplement (10% v/v; Sigma-Aldrich #M0678) and 50 μg/mL Hygromycin B (ThermoFisher #10687010) or liquid 7H9 media (Difco #271310) supplemented with Middlebrook OADC growth supplement (10% v/v), 0.05% Tween 80 (Sigma-Aldrich #P1754), and 50 μg/mL Hygromycin B.

Single cell preparations of these bacteria were prepared and stored as single-use aliquots at −80°C. Briefly, bacteria were grown at 33°C in 50 mL 7H9 supplemented with 10% OADC (Sigma-Aldrich #M0678), 0.05% Tween-80 (Sigma-Aldrich #P1754), and 50 μg/mL hygromycin B (Invitrogen #10687010) (7H9 Complete). Once cultures reach OD_600_ 0.55–0.8, they are spun down at 4600 rcf for 15 min and resuspended in 5 mL PBS-T (1× PBS with 0.05% tyloxapol (Sigma-Aldrich #T8761)) and bring to 25 mL total in PBS-T. They are spun and washed 2× in 25 mL PBS-T each time and then resuspend in 2 mL of 7H9 with 10% OADC (Freezing 7H9) and split into 250 uL aliquots and homogenize each 10× using a 1 mL syringe and 27G needle (BD #309623). Next, a soft spin at 770 rcf for 1 min is done to pellet larger clumps and the supernatants are collected and then push the pooled supernatants through a 5 μm filter (Millipore #SLSV025LS) using a 10 mL syringe. The suspension is collected in 1.5 mL microfuge tubes and spun at 10000 rcf for 5 min. Final resuspension of pellet is done in freezing 7H9 and aliquoted into single use aliquots and concentration is calculated by fluorescent bacteria on a hemocytometer and by colony forming units on selective media.

#### THP-1 culture

THP-1 (ATCC TIB-202) cells were sourced from the Duke Cell Culture Facility and tested for mycoplasma prior to receipt. Cells are cultured in RPMI-1640 (Sigma-Aldrich #R8758) supplemented with glucose (Sigma-Aldrich #G8769), HEPES (Gibco #15630), sodium pyruvate (Gibco #11360) and 10% non-heat inactivated FBS (Sigma-Aldrich #F2442) in T-75 flasks (CellStar #658170) in a 37°C incubator with 5% CO_2_. Cells were cultured for no greater than 10–12 passages prior to use.

### METHOD DETAILS

#### CFU assays

Colony forming unit assays were conducted by complete homogenization of whole adult zebrafish after euthanasia by tricaine overdose and external cleansing of the skin using 70% ethanol. A single 6.5 mm ceramic bead (Omni #19–682) was added to in a pre-filled bead mill tube containing 2.8 mm stainless steel beads (Sigma-Aldrich #Z763829-50EA) and was homogenized on a bead mill (MP Bio #116004500) for a single 25 s interval at 5 m/s. Lysate was plated on 7H10 plates supplemented with 10% OADC, hygromycin B (50 μg/mL), amphotericin B (Gibco #15290–026) (10 μg/mL), and polymyxin B (Cayman Chemical #14157) (25 μg/mL). Lysate was plated in serial 1:10 dilutions up to 10^−5^. Cultures were grown for 10–14 days prior to counting visible colonies. Where possible (due to contamination inherent to the assay), confirmatory counting was performed at 21 days after plating to capture slow-growing colonies. Plates displaying overt contamination that occluded colony growth were excluded from further analysis.

#### Microinjection of TDM and mycobacteria

Bacterial infections were performed as described previously.^44^ In brief, 2 dpf larvae were anesthetized in tricaine and injected with ~50–150 fluorescent bacteria along the trunk into a developmentally undefined peri-notochordal space lying between the somatic muscle layers, allowing the injection bolus to spread along the anterior-posterior length of the fish and establishing a largely localized infection in the avascular trunk.

Microinjection of TDM has been described in previous work. In brief, trehalose 6,6′-dimycolate from *Mycobacterium bovis* (TDM, Sigma-Aldrich #T3034) was resuspended and stored in 2:1 v/v chloroform:methanol at 1 mg/mL. Prior to use, the liquid was evaporated under vacuum and resuspended in incomplete Freund’s adjuvant (IFA, Sigma-Aldrich #F5506) at 2 mg/mL. Larvae were anesthetized in tricaine and injected with approximately 10–20 nL of TDM/IFA or IFA along the trunk. The droplets coalesce into spheres shortly after injection and remain in place for the duration of the experiment. Larvae were then roused in E3 medium supplemented with PTU and allowed to continue development at 28.5°C.

#### Establishment of transgenic lines

Transgenic lines were established using tol2 transgenesis via the tol2kit^119^ and constructed by Gateway cloning.

The p5e irg1 construct was generated by restriction digestion of irg1-pTol2linkerswitch^41^ (a gift from Christopher Hall) with FseI and XmaI and then blunted using T4 DNA polymerase (NEB #M0203S) per the manufacturer’s instructions. Simultaneously, p5e MCS^119^ PCR linearized using inverted T3 and T7 promoter primers (5′- CCCTATAGTGAGTCGTATTAC-3′, 5′- TCCCTTTAGTGAGGGTTAA T-3′), digested with DpnI and PCR purified. These fragments were then ligated using T4 DNA ligase (NEB # M0202S) to generate p5e irg1. This plasmid was then recombined with pME tdTomato (Addgene #135202), p3e ubb pA (Addgene #188702), and pDEST tol2 ubb pA (Addgene #188701) by Gateway cloning (ThermoFisher #12538120) to generate the pTol2 irg1:tdTomato construct that was then injected into single cell embryos alongside 15 ng/μL tol2 mRNA^97,120^ in 13 Tango buffer (ThermoScientific #BY5). Candidate founders were selected based on fluorescence at 3 dpf, raised to adulthood, and outcrossed to *AB to establish the line, which transmits at 50% frequency, suggesting a single insertion locus and has exhibited stable expression over 6 generations.

Tg(*irg1*:*VIVIT-tdTomato*^*xt38*^), in which the inhibitory peptide VIVIT conjugated to the fluorescent protein tdTomato is expressed strictly in macrophages, was constructed by recombination of p5E irg1 (Addgene #188698), pME VIVIT NS (Addgene #188699), p3E tdTomato (Addgene #188700), and pDEST tol2 Ubb pA (Addgene #188701). Reactions were incubated at equimolar ratios overnight in a 25°C thermocycler with heated lid, with volumes calculated using the provided “LR Ratios Calculator” Excel document. The *irg1* promoter was first described by Sanderson et al. as a macrophage-specific inducible promoter, but our lab has found that this element often drives basal expression in macrophages as well, likely in an insertion-site-dependent manner.

The middle element, pME VIVIT NS was constructed by a synthetic templated PCR after annealing. Two oligonucleotides from Integrated DNA Technologies (IDT) were annealed by heating to 95°C and then slowly cooled to room temperature (sense: 5′-GCC ATCATGGCAGGACCACACCCGGTGATTGTTATCACTGGACCACATGAGGAG-3′, anti-sense: 5′-CTCCTCATGTGGTCCAGTGATAACAATCACCGGGTGTGGTCCTGCCATGATGGC-3′). This was then used as a template for PCR using two primers to add the attB1 and attB2 sites required for Gateway recombination into pDONR 221 (forward: 5′-GGGGACAAGTTTGTACAAAAAA GCAGGCTGCCATCATGGCAGGACC-3′, reverse: 5′- GGGGACCACTTTGTACAAGAAAGCTGGGTACTCCTCATGTGGTCCAGTG-3′). This PCR product was then column purified and recombined into pDONR 221 using BP Clonase II (ThermoFisher #11789020) to generate pME VIVIT NS (no stop) (Addgene #188699). Constructs were verified by either Sanger sequencing or whole plasmid sequencing from Plasmidsaurus and have been submitted to Addgene, which provides additional whole plasmid sequencing verification.

Genotyping to differentiate the *irg1*:*tdTomato*^*xt40*^ and *irg1*:*VIVIT-tdTomato*^*xt38*^ lines can be performed where necessary (either for intentional experimental blinding or due to incidental mixing of fish during husbandry or experimentation) by PCR and gel electrophoresis. Primers (5′- GATTTAGGTGACACTATAGATTCAGAGCTCGCACAGG-3′,5′- ATCTCGAACTCGTGGCC-3′) amplify across the 3′ end of the irg1 promoter and into the 5′ end of the tdTomato insert. VIVIT + fish display a 236 bp band while tdTomato-only fish display a 163 bp band. No band is seen in sibling fish lacking an *irg1* transgene.

#### Mutation via CRISPR/Cas9

Generation of mutants in *card9*, *nfatc2a*, and *nfatc3a* was performed as described previously.^121^ Briefly, the oligonucleotides produced by CRISPRscan were utilized as a PCR template paired with the common sgRNA tail oligo (5′-AAAAGCACCGACTCGGTGC CACTTTTTCAAGTTGATAACGGACTAGCCTTATTTTAACTTGCTATTTCTAGCTCTAAAAC-3′). These were mixed at equimolar ratios (5 μL each from 10 μM stocks) into a standard Q5 (NEB #M0491S) reaction mixture containing 23 concentration of dNTPs (NEB #N0447S) and thermocycled using the following parameters: 98°C – 30 s, (98°C – 5 s, 45°C – 30 s, 72°C – 15 s) × 24, 72°C – 5 min, 4°C – hold. This product was then PCR purified using a commercial kit by the manufacturer’s instruction (Macherey-Nagel #740609). This product was then used in an *in vitro* transcription reaction using the NEB T7 HiScribe kit (NEB #E2040S) with the following adjustments: 17 μL template, 2 μL GTP, 2 μL CTP, 2 μL ATP, 2 μL UTP, 2 μL enzyme, 3 μL buffer and left to react overnight at 37°C. This was then purified using the Monarch Total RNA Miniprep kit (NEB #T2010S). RNA was diluted to 500 ng/μL in TE and stored at −80°C until use. On the morning of injection, 1 μL of RNA was added to 1 μL of 63 μM recombinant Cas9 protein (IDT DNA #1081059) in 1× Tango buffer (ThermoFisher #BY5). This mixture was then injected into single cell embryos and these were then either used directly for experiments or raised to adulthood to be screened as potential founders. Alleles were identified by outcrossing of mosaic adults to wild-type *AB and Sanger sequencing of F_1_ adults. DNA extraction was conducted by cellular lysis in 50 mM sodium hydroxide as described previously.^122^ Briefly, either adult zebrafish tail fins or whole larvae were collected in 50 mM NaOH in H_2_O and lysed at 98°C for 12 min in a thermocycler and then neutralized by 1:10 addition a solution of 1M Tris-HCl (pH 8) in 10× TE (100 mM Tris, 10 mM EDTA). This solution was then directly used as the template for downstream PCR reactions.

The allele *card9*^*xt31*^ was generated by injection of a single guide RNA into single-cell embryos (guide sequence: 5′-TAATACGACTCACTATAGGGCAAGGTGCTGAGCAGCGGTTTTAGAGCTAGAA-3′). We identified an allele containing a 28 bp insertion, resulting in an immediate downstream frameshift leading to a premature termination codon at amino acid 59 (with missense mutations beginning at amino acid 47). Genotyping was performed using high-resolution melt analysis (HRMA) using the MeltDoctor Master Mix (Applied Biosystems #4415450) with primers flanking the sgRNA site (5′- CCTTATCTGAGACAGTGCAAGGTGC-3′, 5′- TTACCAACTTTGCGGCGTCTG-3′). Amplification for Sanger sequencing was performed using primers (5′- GTTTTCCCAGTCAC GACCGAATGCTTCTCATCAAGACC-3′, 5′- CGAATGCTTCTCATCAAGACC-3′

The allele *nfatc2a*^*xt69*^ was generated by simultaneous injection of two neighboring guide RNAs to increase odds of a larger intervening deletion (guide sequences: 5′-TAATACGACTCACTATAGGGCTGCGAGAACGGGCCACGTTTTAGAGCTAGAA-3′, 5′-TAA TACGACTCACTATAGGCAGCCCGTCGCCCCACGGGTTTTAGAGCTAGAA-3′). We identified a mutation consisting of a complex, bipartite insertion/deletion leading to a net 4 bp insertion and frameshift leading to a premature termination codon at amino acid 272 (of 894, prior to the DNA binding domain). Genotyping can be performed by one of two distinct restriction digest-based methods. The original method was performed by restriction digest of the ~500 bp PCR product produced by the listed sequencing primers (5′-TAG AAGGCACAGTCGAGGCTCGAGGCTTTCTGGAGACCTCTGTCC-3′, 5′-TGACACACATTCCACAGGGTCTCTAGAGGTTTGCCCTTCATATCCTGC-3′, underlined portion base pairs with the genomic sequence); digestion was with PflMI (NEB #R0509) directly in the PCR reaction mixture. PCR was performed using LongAmp Taq (NEB #M0323) strictly for reasons of buffer compatibility with the restriction enzyme. Digestion was carried out for ≥3h at 37° in the presence of rSAP (NEB #M0371) to minimize background. Sanger sequencing was conducted on undigested PCR products using the vendor (Eton Biosciences) supplied “BGH Reverse” primer (5′-TAGAAGGCACAGTCGAGG-3′) corresponding to the appended 5′ tail of the forward PCR primer.

The second method utilizes a separate set of primers (5′-CCTCTATGCAAACGCACCTACG-3′, 5′-GTGATGCTCCTTGTGGCCA C-3′) to generate a 102–106 bp PCR product spanning the mutation site. This PCR is performed in 20 μL reaction volumes using Taq polymerase (NEB #M0285L) (again, for reasons of buffer compatibility) and 1 μL MwoI (NEB #R0573L) is added directly to the reaction mixture after thermocycling, which is then incubated at 60°C for 1 h. The reaction is then visualized on a 2–3% agarose gel impregnated with SYBR Safe dye. In our hands, this second method is faster, easier, more robust, and more cost-effective. In both cases, the wild-type product is unable to be cut (single larger band) while the mutant is cleaved into two similarly sized smaller bands (a slightly hazy “single” lower band); the heterozygotes are differentiated by the presence of both bands. Confirmatory Sanger sequencing was performed as needed.

The allele *nfatc3a*^*xt59*^ was generated using an individual sgRNA (5′-TAATACGACTCACTATAGGGCAGTTTGCAGTAGTCATGTTTTAGAGCTAGAA-3′) and a mutation was identified containing a 22 bp deletion leading to a premature termination codon at the 8^th^ amino acid (of 1074). The allele was identified by PCR amplification and Sanger sequencing using F: 5′-GTTTTCCCAGTCACGAC CAGAAGGTCGAGCAGTTTGG-3′ and R: 5′-AACGTGTTTCGCCTTTGC-3′. Sequencing used the “M13F(−40)” primer supplied by the vendor (Eton Biosciences) (5′-GTTTTCCCAGTCACGAC-3′). Genotyping was routinely conducted by high-resolution melt analysis (HRMA) using the MeltDoctor Master Mix (ThermoFisher #4415450) with primers flanking the sgRNA site (5′-AAAGAGTCGGTGTACATAGACGGG-3′, 5′-CGAAGATCAGTCTGAAGTCCAGC-3′).

#### Crispant assays

To generate mosaic knockouts in genes of interest, we synthesized sgRNAs targeting the first exon of the respective genes. For *nfatc2a* we used 5′-TAATACGACTCACTATA**GGTCAGTCAGGTGAACTGTC**GTTTTAGAGCTAGAA-3′ and for *nfatc3a* we used 5′-TAATACGACTCACTATA**GGTAGAGGCACTGACCTGCG**GTTTTAGAGCTAGAA-3′. For prospective genotyping of these alleles, we used HRMA to assess approximate editing efficiency; this can only act as a rough proxy due to limitations and feasibility of exhausting genetic analysis of these mosaic larvae. For *nfatc2a*, we used the following primers: 5′-CTCTTTTTACGGCGAAAAAGCTG C-3′, 5′-GAAACAAACCTTGAAGTCCTGTTTGG-3′. For *nfatc3a* we used: 5′-AAAGAGTCGGTGTACATAGACGGG-3′, 5′-CGAAGATCAGTCTGAAGTCCAGC-3′. We had already begun generating the future stable alleles *nfatc2a*^*xt69*^ and *nfatc3a*^*xt59*^ and used these sgRNAs to increase our likelihood of introducing a functional mutation in these genes and to normalize target location and sgRNA number.

#### Adult zebrafish infection

Both male and female zebrafish were used in approximately equal proportion throughout. Fish were anesthetized in 120 μg/mL tricaine. Single cell aliquots of *M*. *marinum* were thawed and diluted in sterile PBS and zebrafish were injected with 10 μL of a solution containing 200–1000 fluorescent bacteria using a back-loaded insulin syringe (BD #08290-3284-38). Zebrafish were maintained in spawning tanks (Aquaneering #ZHCT100) with daily water changes and feeding. Water taken from the primary zebrafish system was used to ensure stable water quality throughout experimentation.

#### CLARITY and confocal microscopy

CLARITY fixation and clearing was conducted as previously described.^67^ In brief, adult zebrafish were euthanized in tricaine, decapitated, and disemboweled. Visceral organs were immersed in an A1P4 CLARITY solution (4% paraformaldehyde (EMS #15710), 1% acrylamide (Bio-Rad #1610140), 0.05% bis acrylamide (Bio-Rad #1610142), 0.0025 g/mL radical initiator (Wako Chemical #VA-044) in 1× final concentration PBS (Corning #46013CM) and nutated at 4°C for 2 days prior to overlay with mineral oil (Fisher Scientific #BP2629) and polymerized at 37°C for 3 h. Hydrogel samples were collected, washed in 1× PBS, and then immersed in clearing solution at 37°C (8% sodium dodecyl sulfate (Bio-Basic #SD8119) in 200 mM boric acid (Sigma-Aldrich #B0394), pH 8.5), which was changed every 2–3 days until samples were optically clear. These samples were washed in 1× PBS supplemented to 0.1% Triton-X (Fisher Scientific #BP151) for two days at 37°C with daily solution changes to remove excess SDS from the tissue. These tissues were then individually placed into black, opaque microcentrifuge tubes and immersed in refractive index matching solution (RIMS) (40 g, Histodenz (Sigma-Aldrich #D2158), 30 mL 20 mM phosphate buffer (4.043 g Na_2_HPO_4_ (VWR #BDH9296), 678.7 mg NaH_2_PO_4_ (Sigma-Aldrich #S9638), 1 L diH_2_O), 0.01% sodium azide (Sigma-Aldrich #71290)) with rotation for at least 24 h prior to imaging.^123^

Imaging was conducted on a spinning disk microscope (Zeiss AxioObserver Z1 connected to an XCite 120 LED Boost with an XLight 2TP, 89North LDI, Hamamatsu C13440 and captured on a Dell Precision Tower 5810 running Windows 10 Enterprise with Metamorph 7.10.5.476) in a MatTek dish (#P35G-1.5-14-C) with optical bottom. Additional RIMS was added to the dish to cover the sample and minimize refraction during imaging. We panned across the proximal surface of the organ bundles to identify granulomas in each individual sample and captured Z-stack images of each of the identifiable granulomas at the maximum possible optical depth in the fish. This is able to capture the majority (but perhaps not all) of the granulomas present in a given fish due to inherent limitations in lens working distance.

All image processing was conducted in FIJI/ImageJ.^104^ In-focus Z planes were identified and processed with the Maximum Intensity Projection function using a Jython macro. These files were saved and then subjected to cropping where the frame was cropped to the vasculature immediately surrounding each granuloma. This distance was unable to be precisely normalized across granulomas due to the differing sizes and shapes of the granulomas themselves as well as the nature of their varying physiological locations. Cropped images were then blinded using the blindrename.pl script.^68^ Images were then opened in ImageJ and vessels were traced using the segmented line tool, added to the Region of Interest (ROI) Manager tool and then measured for distance in pixels. Total length was then converted to microns based on the conversion factor provided by the microscope (1 px = 0.6552 μm). Resulting .csv files were processed in Excel to remove unnecessary tag information from files names and then all subsequent analysis was performed in R using RStudio (citations).

#### qRT-PCR

THP-1 cells were transdifferentiated into macrophage-like cells using 50 ng/mL PMA (phorbol 12-myristate-13-acetate) (Sigma-Aldrich #P148), seeded in 24 well cell culture treated plates at a concentration of 5 × 10^5^ cells/mL and incubated at 37°C/5%CO_2_ for 48hr. After that the PMA media was changed using complete RPMI 1640 media and incubated at 37°C/5%CO_2_ for 24hr (rest day). Then the cells were exposed to 0.5 mL of gamma-irradiated *Mtb* (BEI #NR-49098) in 25% glycerol (Sigma-Aldrich #G7757) diluted in RPMI-1640 at a final concentration of 1 mg/mL. Cells were spun at 100 rcf for 5 min and incubated at 37°C/5%CO_2_ for 8hr.

Cells then had media removed and were washed once with 1× PBS. After removing the PBS, 300 μL of Trizol was add and cells were vigorously resuspended and moved into 1.5 mL microfuge tubes. RNA extraction was conducted by addition of an equal volume of 1× TE (Sigma-Aldrich #T9285) and 100 μL of chloroform (EMD Millipore #CX1055). After spinning at 17,000 rcf for 30 min at 4°C, the upper aqueous layer was transferred to another tube, and 100 μL of 24:1 chloroform:isoamyl alcohol (Sigma-Aldrich #25666) was added. The tubes were then shaken by hand and spun for another 30 min at 17,000 rcf at 4°C. The top aqueous layer was removed and final cleanup was done using the RNA Cleanup Kit (NEB #T2040L) per the manufacturer’s instructions.

cDNA synthesis was performed using the LunaScript RT SuperMix Kit (NEB #E3010L) by the manufacturer’s instructions. RT-PCR was performed using the Luna Universal qPCR Master Mix (NEB #M3003L) in an Applied Biosystems 7500 Fast (ThermoFisher #4351106) per the manufacturer’s instructions. Final calculations were conducted in R.

#### ELISA

Cells were cultured identically to previous, except they were plated in 96 well cell culture treated plates and exposed to gamma-irradiated *Mtb* for a total of 24 h to facilitate VEGF production and secretion. Supernatants were collected and spun down and then the upper layer was collected for further analysis. ELISA was performed according to the manufacturer’s instructions (R&D Systems #DY293B). Absorbance was read on an Agilent Synergy LX plate reader.

#### Immunofluorescence

THP1 cells were plated on 4-well chamber slides and differentiated with PMA at 50 ng/mL for 48 h. Media was then replaced with fresh RPMI-1640 and cells were allowed to rest for 24 h prior to further stimulation. Cells were then treated by addition of 1 mg/mL final concentration gamma-irradiated *Mtb*, 40 μM INCA-6 (Cayman Chemicals #21812), and/or vehicle controls (25% glycerol in PBS or DMSO (Fisher Scientific #BP337), respectively). Cells were then incubated at 37°C, 5% CO_2_ for 8 h and then fixed in 4% PFA in 1× PBS for 20 min. Cells were then washed twice in 0.25% NH_4_Cl (Sigma-Aldrich #254134) (to neutralize, rinsed in PBS, blocked in 2.5% donkey serum (Fisher Scientific #50413253) in 1× PBS for at least 20 min, and then incubated in primary antibody overnight at 4°C. Cells were then rinsed, secondary antibody was added and cells were again incubated overnight at 4°C. After 5× rinses in PBS, cells were dipped in diH_2_O and mounted in DAPI Fluoromount-G (SouthernBiotech Cat #: 0100–20), which was allowed to set overnight at RT in the dark. Slides were either stored at 4°C in the dark prior to visualization or visualized immediately.

Images shown in the figures were digitally adjusted for brightness and contrast in FIJI/ImageJ^101^ and all adjustments were applied uniformly across the images within an experiment. All quantitation was performed based on the unadjusted brightness and contrast values and thresholded to better capture positive signal and eliminate the background fluorescence ubiquitous in these images.

Zeiss filter sets used were:

- Filter Set 50 (Cy5, Alexa Fluor 647)
- Filter Set 47 (CFP)
- Filter Set 38 (GFP, Alexa Fluor 488)
- Filter Set 43HE (tdTomato, Alexa Fluor 555)
- Filter Set 46 (YFP)
- Filter Set 49 (DAPI)

#### Lentivirus construction

We sought to generate lentiviruses able to target multiple single guide RNAs to the same gene to maximize overall mutation rate and allow us to conduct experiments in mixed pools of heterogeneous cells, to minimize functional passage number. We therefore adopted a hybrid approach, inserting the sgRNA targeting array and hUbC promoter from Kabadi et al. 2014 (Addgene #53190, a kind gift from Charles Gersbach) into the NotI/XbaI site of the lentiCRISPRv2 plasmid from Sanjana et al. 2014 (Addgene #52961, a kind gift from Feng Zhang), creating a hybrid plasmid that simultaneously expressed Cas9, the puromycin resistance marker, and up to 4 single guide RNAs from a single plasmid.

This resulting transfer empty vector (pLV hUbC-Cas9-P2A-Puro_BsmBI-sgRNA-BsmBI, Addgene #188703) was digested with Esp3I FastDigest (ThermoFisher #FD0454) precisely as previously described^73^ in the presence of equal *masses* (~200 ng each) of constituent sgRNA expression plasmids driven from mU6, hU6, 7SK, or hH1 RNA pol III promoters, ligated with T4 ligase (NEB #M0202S), and cloned into NEB Stable (NEB #C3040H) cells. Resulting plasmids were screened by restriction digestion and full plasmid sequencing.

Single guide RNA expression plasmids were cloned from phU6-gRNA, pmU6-gRNA, ph7SK-gRNA, and phH1-gRNA as described previously.^73^ The guide sequences for both NFATC2 and the safe targeting loci were chosen from the a database of available guides and safe loci in the human genome to model the DNA damage response from sgRNA targeting without overt toxicity or phenotypic changes.^75^ These plasmids were purified and used in subsequent steps.

The appropriate lentivirus transfer plasmid was transfected into HEK293T cells alongside pMD2.G (Addgene #12259) and psPAX2 (Addgene #12260) (both kind gifts from Didier Trono) (plus sfGFP-C1 to mark transfected cells, Addgene #54579, a kind gift of Michael Davidson & Geoffrey Waldo) in a 4:3:1(:0.5) mass ratio using TransIT-Lenti reagents (Mirus Bio #MIR-6603).^98^ Supernatants were collected 48 h post transfection and immediately used to transduce THP-1 cells in the presence of 8 μg/mL polybrene (Sigma-Aldrich #TR-1003-G). Approximate titer was determined by infecting additional HEK293T cells with varying dilutions of the supernatant.

#### THP-1 transduction

THP-1 cells were seeded in complete RPMI-1640 media supplemented with 8 ng/mL polybrene (Sigma-Aldrich #TR-1003-G) in two non-treated six-well plates at a concentration of 1 × 10^6^ cells/mL in each well. One six-well plate was infected with 1mL of pLV-ST and the other with pLV-NFATC2. The lentivirus infected THP-1 cells were spun at 1500 rcf/2 h/22°C, gently resuspended and incubated at 37°C/5%CO_2_ for 72hr. Transduced cells were selected with 2 μg/mL puromycin (Sigma-Aldrich #P4512) for 48 h and then kept in complete RPMI-1640 with 1 μg/mL puromycin until time of assay.

#### Immunofluorescence analysis

To capture differences in VEGF expression across different experimental conditions, we programmatically blinded a subset of images from each experimental condition using blindrename.pl^68^ or our in-house Python translation and, using the “Cell Counter” plugin in FIJI/ImageJ, we marked each nucleus (as a proxy for cell number), each cell that visually expressed VEGFA at a minimum/maximum bit value of 100/1500, cells that had nuclear translocation of NFATC2, and, when applicable, Cas9 expression. These values were exported and subsequently processed in R.

### QUANTIFICATION AND STATISTICAL ANALYSIS

All assays were performed under experimental blinding. For all assays where the genotype or experimental condition of the fish was apparent to the experimenter during data gathering (for instance, experiments in adults and the VIVIT assays in larvae), the resulting images were computationally blinded prior to analysis with either blindrename.pl^68^ or an in-house Python translation (available at https://doi.org/10.5281/zenodo.6816429). For assays where the genotype is unknown (in-cross of heterozygotes experiments for *card9*, *nfatc2a* in the larvae), blinding was inherent in the design of the experiment and genotypes were matched to the individual fish *post hoc*.

Statistical analysis was performed using R 4.2.2 within the latest version of RStudio IDE.^99,100^ Graphing was performed using ggplot2.^106,108^ All statistical tests performed and the resulting significance values are indicated in figures and figure legends.

#### R

R (version 4.2.2, “Innocent and Trusting”) was accessed via RStudio (“Elsbeth Geranium” version 2022.12.0) on macOS 12.6.1 “Monterey”.^99,100^

Analysis and visualization were conducted using in-house workflows developed for these data types. Data analysis required use of dplyr,^109^ reshape,^115^ and FSA.^117^ Graphs utilized ggplot2,^106,108,107^ gghighlight,^110^ ggbeeswarm,^111^ ggsignif,^112^ scales,^113^ extrafont,^114^ and RColorBrewer.^116^ All scripts and related materials are available in the accompanying data release at Zenodo (https://doi.org/10.5281/zenodo.6816429).

#### FIJI/ImageJ

Image analysis was conducted using the FIJI^101,102^ expansion of ImageJ.^104,103^ Analysis pipelines were written in Jython (v.2.7.2)^105^ and executed within the ImageJ Jython interpreter. All scripts are provided via Zenodo at the doi listed above.

## Supplementary Material

1

2

3

## Figures and Tables

**KEY RESOURCES TABLE:** 

REAGENT or RESOURCE	SOURCE	IDENTIFIER

Antibodies		

polyclonal goat anti-human VEGFA antibody	R&D Systems	Cat# AF-293; RRID: AB_354450
monoclonal mouse anti-Cas9 antibody	Cell Signaling	Cat# 7A9-3A3
Normal Goat IgG Control	R&D Systems	Cat# AB-108-C; RRID: AB_354267
rabbit anti-human NFATC1 serum (against NH_2_-CVSPKTTDPEEGFPRGLGA, residues 210 to 227)	Lyakh et al.^85^; Symes et al.^47^	#801
rabbit anti-human NFATC2 serum (against NH_2_-CSPPSGPAYPDDVLDYGLK, residues 53 to 70)	Lyakh et al.^85^; Symes et al.^47^	#1777
rabbit anti-human NFATC3 serum (against NH_2_-DLQINDPEREFLERPSRDHL, residues 130 to 149)	Lyakh et al.^85^; Symes et al.^47^	#1689
rabbit anti-human NFATC4 serum (against NH_2_-GRDLSGFPAPPGEEPPA, residues 886 to 902)	Lyakh et al.^85^; Symes et al.^47^	#889
rabbit anti-human NFATC4 serum (against NH_2_-CDSKVVFIERGPDGKLQWEE, residues 614 to 632)	Lyakh et al.^85^; Symes et al.^47^	#890
rabbit anti-human pan-NFAT serum (against NH_2_-SDIELRKGETDIGRKNTRC)	Lyakh et al.^85^; Symes et al.^47^	#796
donkey anti-goat IgG Alexa Fluor 647	ThermoFisher	Cat# A-21447; RRID: AB_2535864
donkey anti-goat IgG Alexa Fluor 555	ThermoFisher	Cat# A-21432; RRID: AB_2535853
donkey anti-rabbit IgG Alexa Fluor 647	ThermoFisher	Cat# A-31573; RRID: AB_2536183
donkey anti-rabbit IgG Alexa Fluor 555	ThermoFisher	Cat# A-31572; RRID: AB_162543
donkey anti-mouse IgG Alexa Fluor 555	ThermoFisher	Cat# A-31570; RRID: AB_2536180
donkey anti-mouse IgG Alexa Fluor 488	ThermoFisher	Cat# A-21202; RRID: AB_141607

Bacterial and virus strains		

*Mycobacterium marinum* M	ATCC	Cat# BAA-535
*Mycobacterium marinum* M / pMSP12:mCerulean	Oehlers et al.^18^	N/A
*Mycobacterium marinum* M / pMSP12:tdTomato	Cambier et al.^96^	N/A
Gamma-irradiated *Mycobacterium tuberculosis* H37Rv	BEI	Cat# NR-49098
NEB 5-alpha Competent *Escherichia coli* (High Efficiency)	NEB	Cat# C2987H
NEB^®^ 10-beta Competent *Escherichia coli* (High Efficiency)	NEB	Cat# C3019H
NEB Stable Competent *Escherichia coli* (High Efficiency)	NEB	Cat# C3040H

Chemicals, peptides, and recombinant proteins		

Trizol	Ambion	Cat# 15596026
MicroAmp Fast Optical 96-Well Reaction Plate with Barcode, 0.1 mL	Applied Biosystems	Cat# 4346906
Spawning Tanks	Aquaneering	Cat# ZHCT100
Baking soda (sodium bicarbonate)	Arm & Hammer	Cat# #426292
Insulin Syringes	BD	Cat# 08290-3284-38
Tuberculin Syringe (27G)	BD	Cat# 309623
SDS, 20%(w/v) solution, 1L	3io-Basic	Cat# SD8119
40% acrylamide	3io-Rad	Cat# 1610140
2% bis-acrylamide	3io-Rad	Cat# 1610142
*Artemia*	Brine Shrimp Direct	Cat# BSEP6LB
Polymyxin B sulfate	Cayman Chemical	Cat# 14157
INCA-6	Cayman Chemical (Roehrl et al.^71^)	Cat# 21812
T-75 Flasks	CellStart	Cat# 658170
Molecular Biology Grade Water	Corning	Cat# 46000CI
10× PBS	Corning	Cat# 46013CM
7H10	Difco	Cat# 262710
7H9	Difco	Cat# 271310
Chloroform	EMD Millipore	Cat# CX1055
16% Methanol-free Paraformaldehyde	EMS	Cat# 15710
Triton X-100	Fisher Scientific	Cat# BP151
Dimethyl sulfoxide (DMSO)	Fisher Scientific	Cat# BP231
Mineral oil	Fisher Scientific	Cat# BP2629
Tween-80	Fisher Scientific	Cat# BP337
Sodium chloride	Fisher Scientific	Cat# S271
1× PBS	Gibco	Cat# 10010–023
Sodium pyruvate	Gibco	Cat# 11360
Amphotericin B	Gibco	Cat# 15290–026
HEPES	Gibco	Cat# 15630
Alt-R^®^ S.p. Cas9 Nuclease V3, 500 mg	IDT DNA	Cat# 1081059
Instant Ocean Sea Salt	Instant Ocean	Cat# SS15-10
Hygromycin B solution	Invitrogen	Cat# 10687010
4-well Cell Culture Slides	MatTek	Cat# CCS-4
35 mm Dish, No. 1.5 Coverslip, 14 mm Glass Diameter, Uncoated	MatTek	Cat# P35G-1.5-14-C
Tris (base)	Millipore	Cat# 648311
Millex-SV 5.0 μm	Millipore	Cat# SLSV025LS
T4 DNA Ligase	NEB	Cat# M0202S
Taq 5× Master Mix	NEB	Cat# M0285L
LongAmp Taq	NEB	Cat# M0323L
rSAP	NEB	Cat# M0371L
Q5 High-Fidelity DNA Polymerase	NEB	Cat# M0491L
Q5 High-Fidelity 2X Master Mix	NEB	Cat# M0492L
Deoxynucleotide (dNTPs) Solution Mix	NEB	Cat# N0447L
XbaI	NEB	Cat# R0145L
DpnI	NEB	Cat# R0176L
XmaI	NEB	Cat# R0180L
PfIMI	NEB	Cat# R0509L
MwoI	NEB	Cat# R0573L
FseI	NEB	Cat# R0588L
NotI	NEB	Cat# R3189L
Total RNA Cleanup Kit	NEB	Cat# T2010S
RNA Cleanup Kit (50 μg)	NEB	Cat# T2040L
6.5mm ceramic beads	Omni	Cat# 19–682
Petri dishes for embryonic zebrafish	Sarstedt	Cat# 83.3902.500
FK506 (tacrolimus)	Selleck Chemicals	Cat# S5003
Methanol	Sigma-Aldrich	Cat# 179337
Ammonium chloride	Sigma-Aldrich	Cat# 254134
24:1 chloroform:isoamyl alcohol	Sigma-Aldrich	Cat# 25666
Sodium azide	Sigma-Aldrich	Cat# 71290
Boric acid	Sigma-Aldrich	Cat# #B0394
Sodium phosphate monobasic monohydrate	Sigma-Aldrich	Cat# D2158
Fetal bovine serum	Sigma-Aldrich	Cat# F2442
Incomplete Freund’s adjuvant (IFA)	Sigma-Aldrich	Cat# F5506
Glycerol	Sigma-Aldrich	Cat# G7757
Glucose solution	Sigma-Aldrich	Cat# G8769
OADC	Sigma-Aldrich	Cat# M0678
Phorbol-12-myristate-13-acetate (PMA)	Sigma-Aldrich	Cat# P148
Tween 20	Sigma-Aldrich	Cat# P1754
1-phenyl-2-thiourea	Sigma-Aldrich	Cat# P7629
RPMI-1640	Sigma-Aldrich	Cat# R8758
trehalose 6-6′-dimycolate (TDM) from *M. bovis*	Sigma-Aldrich	Cat# T3034
Tyloxapol	Sigma-Aldrich	Cat# T8761
100× Tris-EDTA (TE)	Sigma-Aldrich	Cat# T9285
Polybrene	Sigma-Aldrich	Cat# TR-1003-G
BeadBug homogenizer tubes with 2.8mm stainless steel beads	Sigma-Aldrich	Cat# Z763829-50EA
Dry fish food	Skretting	Cat# GEMMA Micro 500
DAPI Fluoromount-G	SouthernBiotech	Cat# 0100–20
Tricaine-S (MS-222)	Syndel	Cat# ANADA 200–226
Brefeldin A Solution (1000X)	ThermoFisher	Cat# 00-4506-51
BP Clonase II	ThermoFisher	Cat# 11789020
LR Clonase II Plus	ThermoFisher	Cat# 12538120
FastDigest Esp3I (IIs class)	ThermoFisher	Cat# FD0454
Calcium chloride	VWR	Cat# BDH9224
Potassium chloride	VWR	Cat# BDH9258
Sodium phosphate dibasic heptahydrate	VWR	Cat# BDH9296
2,2′-Azobis[2-(2-imidazolin-2-yl) propane]dihydrochloride	Wako Chemicals	Cat# VA-044
Magnesium chloride	Ward’s Scientific	Cat# 470301

Critical commercial assays		

MeltDoctor HRM Master Mix	Applied Biosystems	Cat# 4415450
Luna Universal qPCR Master Mix	NEB	Cat# M3003X
Human VEGF DuoSet ELISA	R&D Systems	Cat# DY293B-05
LunaScript RT SuperMix Kit	NEB	Cat# E3010L
HiScribe^™^ T7 High Yield RNA Synthesis Kit	NEB	Cat# E2040S

Experimental models: Cell lines		

THP-1 monocytic cells	ATCC	Cat# TIB-202
HEK-293T	ATCC	Cat# CRL-2316

Experimental models: Organisms/strains		

*Danio rerio* strain *AB	ZIRC	ID# ZDB-GENO-960809-7
*Tg(irg1:tdTomato* ^*xt40*^ *)*	This work	N/A
*Tg(irg1*: *VIVIT-tdTomato*^*xt38*^*)*	This work	N/A
*Tg(kdrl:eGFP* ^*s843*^ *)*	Jin et al.^39^	N/A
*TgBAC(vegfaa:eGFP* ^*pd260*^ *)*	Karra et al.^43^	N/A
*nfatc3a* ^*x59*^	This work	N/A
*card9* ^*xt31*^	This work	N/A

Oligonucleotides		

See Table S1		

Recombinant DNA		

p5E irg1	Addgene (Sanderson et al.^41^)	Cat# 188698
pME VIVIT NS	This work; Addgene	Cat# 188699
p3E tdTomato	Addgene (Walton et al.^97^)	Cat# 188700
pDEST tol2 Ubb pA	Addgene (Walton et al.^97^)	Cat# 188701
pME tdTomato	Addgene (Oehlers et al.^18^)	Cat# 135202
p3e Ubb pA	Addgene (Walton et al.^97^)	Cat# 188702
pTol2 irg1:VIVIT-tdTomato	This work	N/A
pTol2 irg1:tdTomato	This work	N/A
pLV hUbC-Cas9-P2A-Puro_BsmBI-sgRNA	This work, derived from (Kabadi et al.^73^; Sanjana et al.^74^); Addgene	Cat# 188703
pLV hUbC-Cas9-P2A-Puro sgRNA αNFATC2	This work, Addgene	Cat# 188704
pLV hUbC-Cas9-P2A-Puro sgRNA αSafe Targeting Loci	This work, Addgene	Cat# 188705
phU6 NFATC2	This work, Addgene	Cat# 188708
pmU6 NFATC2	This work, Addgene	Cat# 188709
p7SK NFATC2	This work, Addgene	Cat# 188710
phH1 NFATC2	This work, Addgene	Cat# 188711
phU6 ST	This work, Addgene	Cat# 188712
pmU6 ST	This work, Addgene #188713	Cat# 188713
p7SK ST	This work, Addgene	Cat# 188714
phH1 ST	This work, Addgene	Cat# 188715
psPAX2	Addgene	Cat# 12260
pMD2.G	Addgene	Cat# 12259
sfGFP-C1	(Pédelacq et al.^98^), Addgene	Cat# 54579

Software and algorithms		

R, 4.2.1	R Core Team^99^	N/A
RStudio, 2022.06 “Spotted Wakerobin”	RStudio Team^100^	N/A
FIJI/ImageJ2, 2.5.0	(Schindelin et al.^101^; Rueden et al.^102^)	N/A
ImageJ, 1.53s	(Girish and Vijayalakshmi^103^; Schneider et al.^104^)	N/A
Python/Jython, 2.7.18	(van Rossum^105^)	N/A
ggplot2, 3.3.5	(Wickham^106,107^; Wickham et al.^108^)	N/A
dplyr, 1.0.9	(Wickham et al.^109^)	N/A
gghighlight, 0.3.3	(Yutani^110^)	N/A
ggbeeswarm, 0.6.1	(Clarke and Sherrill-Mix^111^)	N/A
ggsignif, 0.6.3	(Ahlmann-Eltze and Patil^112^)	N/A
blindrename.pl, 1.0	(Salter^68^)	N/A
scales, 1.2.0	(Wickham and Seidel^113^)	N/A
extrafont, 0.18	(Chang^114^)	N/A
reshape, 0.8.9	(Wickham^115^)	N/A
RColorBrewer, 1.1–3	(Neuwirth^116^)	N/A
FSA, 0.9.3	(Ogle et al.^117^)	N/A
HRM Software, 3.0.2	ThermoFisher	N/A

Other		

MP Bio FastPrep 24 Classic (Bead Mill)	MP Bio	Cat# 116004500
Applied Biosystems 7500 Fast Real-Time PCR System	ThermoFisher	Cat# 4351106
Nikon Stereomicroscope	Nikon	Cat# SMZ745
Nikon High Intensity Illuminator	Nikon	Cat# NI-150
Eppendorf Femtojet 4×	Eppendorf	Cat# 5253000025
Precision Plant Growth Chamber, 504 L	ThermoFisher	Cat# PR505755L
Zeiss AxioObserver Z1	Zeiss AxioObserver Z1	N/A
X-Cite 120Q	Excelitas	Cat# 12–63000
Cryostat	Leica	Cat# CM1860

## Highlights

- NFAT signaling induces VEGFA in macrophages downstream of a bacterial glycolipid
- NFATC2 is required for angiogenesis induced by mycobacterial trehalose 6,6′-dimycolate
- Macrophage-specific inhibition of NFAT blocks angiogenesis during infection
- *In vivo*, macrophage-specific inhibition of NFAT reduces mycobacterial burden
