# Supplementary material for: Macrophage NFATC2 mediates angiogenic signaling during mycobacterial infection
Source: Cell Rep. Author manuscript; Available in PMC 2023 Jan 27. (PMC9880963; doi:10.1016/j.celrep.2022.111817)
Supplement: 1 [file NIHMS1857926-supplement-1.pdf]

Cell Reports, Volume *41*

Supplemental information

Macrophage NFATC2 mediates angiogenic signaling during mycobacterial infection

W. Jared Brewer, Ana María Xet-Mull, Anne Yu, Mollie I. Sweeney, Eric M. Walton, and David M. Tobin

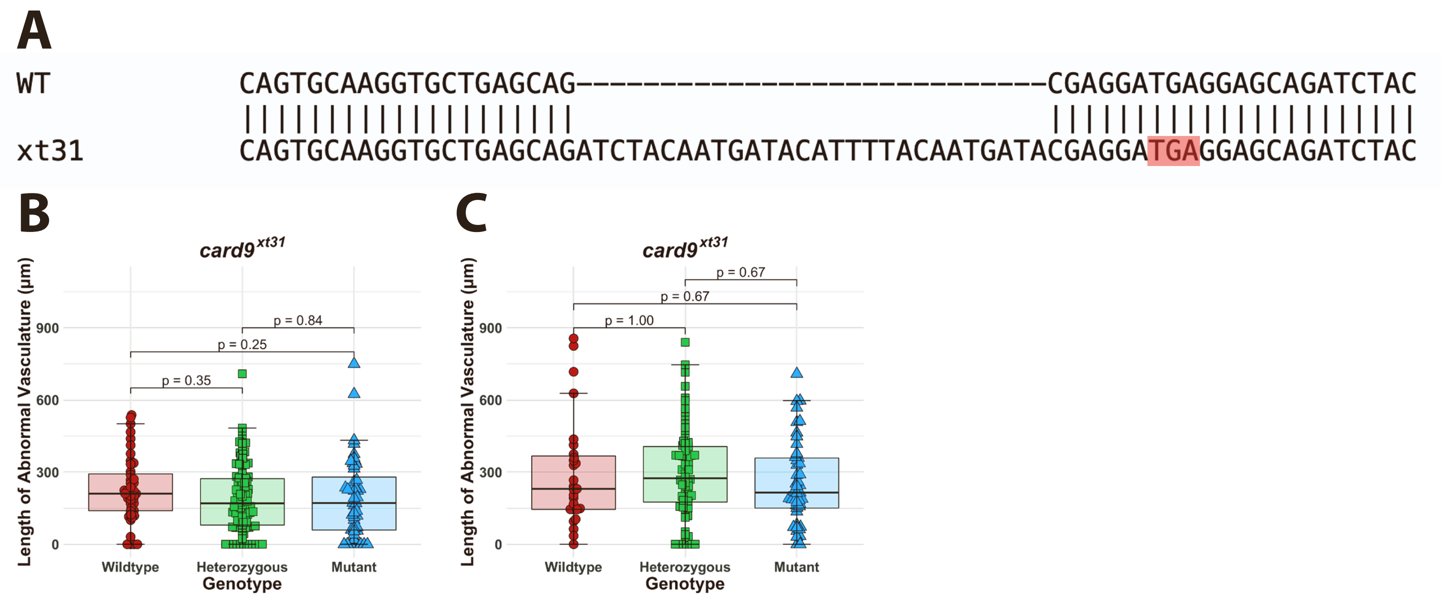
A
WT CAGTGCAAGGTGCTGAGCAG----------------------------CGAGGATGAGGAGCAGATCTAC
xt31 CAGTGCAAGGTGCTGAGCAGATCTACAATGATACATTTTACAATGATACGAGGATGAGGAGCAGATCTAC
B
card9xt31
Length of Abnormal Vasculature (µm)
p = 0.84
p = 0.25
p = 0.35
Wildtype
Heterozygous
Mutant
Genotype
C
card9xt31
p = 0.67
p = 0.67
p = 1.00

Supplemental Figure 1, related to Figure 2. Generation and Analysis of *card9* Mutants.

A. Sequence alignment of the Sanger-sequencing determined wildtype sequence (100% match to reference) and *card9*^{xt31} genomic sequence displaying the 28 base pair insertion and location of the premature termination codon (highlighted in red).
B. Additional replicate of *Mm* infection of larvae from an incross of *kdrl*:*eGFP*; *card9*$^{xt31/+}$ zebrafish (see Figure 2A, 2B). n = 27 wildtype, 70 heterozygotes, 45 homozygous mutants. No statistically significant differences are observed across the groups by Dunn's Kruskal-Wallis multiple comparisons test with Holm error correction.
C. Additional replicate of *Mm* infection of larvae from an incross of *kdrl*:*eGFP*; *card9*$^{xt31/+}$ zebrafish (see Figure 2A, 2B). n = 44 wildtype, 97 heterozygotes, 48 homozygous mutants. No statistically significant differences are observed across the groups by Dunn's Kruskal-Wallis multiple comparisons test with Holm error correction.

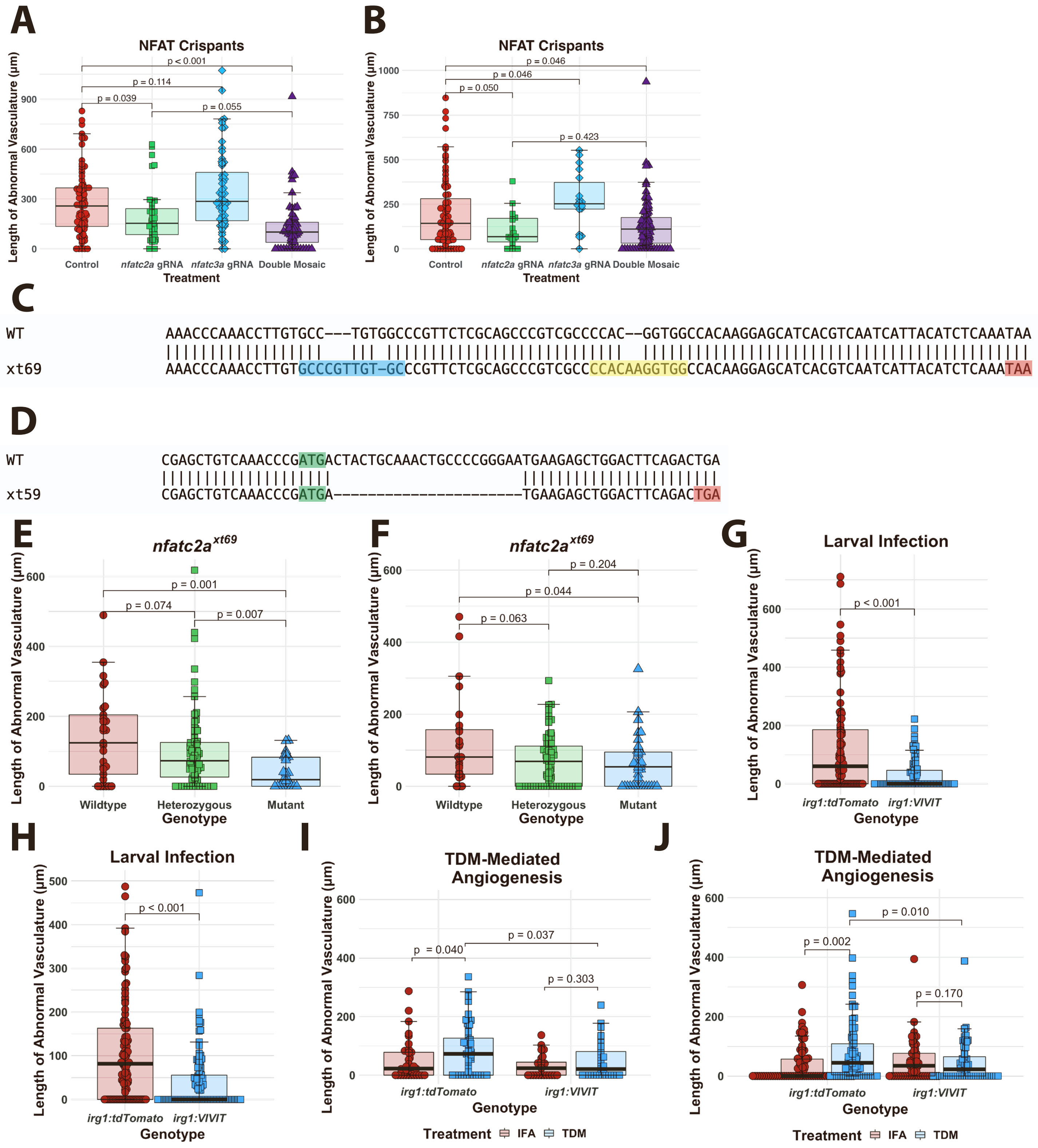
A
NFAT Crispants
p < 0.001
p = 0.114
p = 0.039
p = 0.055
Length of Abnormal Vasculature (µm)
Control
nfatc2a gRNA
nfatc3a gRNA
Double Mosaic
Treatment
B
NFAT Crispants
p = 0.046
p = 0.046
p = 0.050
p = 0.423
Length of Abnormal Vasculature (µm)
Control
nfatc2a gRNA
nfatc3a gRNA
Double Mosaic
Treatment
C
WT AAACCCAAACCTTGTGCC---TGTGGCCCGTTCTCGCAGCCCGTCGCCCCAC--GGTGGCCACAAGGAGCATCACGTCAATCATTACATCTCAAATAA
xt69 AAACCCAAACCTTGTGCCCGTTGT-GCCCGTTCTCGCAGCCCGTCGCCCCACAAGGTGGCCACAAGGAGCATCACGTCAATCATTACATCTCAAATAA
D
WT CGAGCTGTCAAACCCGATGACTACTGCAAACTGCCCCGGGAATGAAGAGCTGGACTTCAGACTGA
xt59 CGAGCTGTCAAACCCGATGA---------------------TGAAGAGCTGGACTTCAGACTGA
E
nfatc2a^xt69
p = 0.001
p = 0.074
p = 0.007
Length of Abnormal Vasculature (µm)
Wildtype
Heterozygous
Mutant
Genotype
F
nfatc2a^xt69
p = 0.204
p = 0.044
p = 0.063
Length of Abnormal Vasculature (µm)
Wildtype
Heterozygous
Mutant
Genotype
G
Larval Infection
p < 0.001
Length of Abnormal Vasculature (µm)
irg1:tdTomato
irg1:VIVIT
Genotype
H
Larval Infection
p < 0.001
Length of Abnormal Vasculature (µm)
irg1:tdTomato
irg1:VIVIT
Genotype
I
TDM-Mediated Angiogenesis
p = 0.040
p = 0.037
p = 0.303
Length of Abnormal Vasculature (µm)
irg1:tdTomato
irg1:VIVIT
Genotype
Treatment IFA TDM
J
TDM-Mediated Angiogenesis
p = 0.010
p = 0.002
p = 0.170
Length of Abnormal Vasculature (µm)
irg1:tdTomato
irg1:VIVIT
Genotype
Treatment IFA TDM

Supplemental Figure 2, Related to Figure 3. Requirement of *nfatc2a* for Infection-associated and TDM-mediated Angiogenesis.

A. Additional replicate of *Mm* infection of larvae from the crispant screen (see Figure 3A). The groups with sgRNA targeting *nfatc2a* show a reduction in the degree of angiogenesis (*nfatc2a* gRNA and Double Mosaic) while the *nfatc3a* sgRNA does not change the degree of angiogenesis. Statistics are from Dunn's Kruskal-Wallis multiple comparisons test with Benjamini-Hochberg adjustment for independent tests. n = 79 control, 37 *nfatc2a*, 60 *nfatc3a*, 59 double mosaic.
B. Additional replicate of *Mm* infection of larvae from the crispant screen (see Figure 3A). The groups with sgRNA targeting *nfatc2a* show a reduction in the degree of angiogenesis (*nfatc2a* gRNA and Double Mosaic) while, in this replicate, the *nfatc3a* sgRNA showed a slight increase in the degree of angiogenesis, although the sample size for that group was relatively small. Statistics are from Dunn's Kruskal-Wallis multiple comparisons test with Benjamini-Hochberg adjustment for independent tests. n = 79 control, 22 *nfatc2a*, 18 *nfatc3a*, 87 double mosaic.
C. Sequence alignment of empirically determined reference *nfatc2a* (which corresponds 100% to the Ensembl reference) and $nfatc2a^{xt69}$. The net mutation is a 4 base pair insertion. The MwoI restriction site is highlighted in blue, the PflMI site is highlighted in yellow, and the premature termination codon is in red.
D. Sequence alignment of empirically determined reference *nfatc3a* (which corresponds 100% to the Ensembl reference) and $nfatc3a^{xt59}$. The net mutation is a 22 base pair deletion. The start codon is highlighted in green and the premature termination codon is in red.
E. Additional replicate of *Mm* infection of larvae from an incross of *kdrl*:*eGFP*; $nfatc2a^{xt69/+}$ zebrafish (see Figure 3C, 3D). Statistics are from Dunn's Kruskal-Wallis multiple comparisons test with Benjamini-Hochberg adjustment for independent tests. n = 29 wildtype, 76 heterozygous, 35 homozygous mutant.
F. Additional replicate of *Mm* infection of larvae from an incross of *kdrl*:*eGFP*; $nfatc2a^{xt69/+}$ zebrafish (see Figure 3C, 3D). Statistics are from Dunn's Kruskal-Wallis multiple comparisons test with Benjamini-Hochberg adjustment for independent tests. n = 25 wildtype, 75 heterozygous, 38 homozygous mutant.
G. Additional replicate of larval infection of *irg1*:*VIVIT-tdTomato*; *kdrl*:*eGFP* and *irg1*:*tdTomato*; *kdrl*:*eGFP* zebrafish (see Figure 4C). Statistics from Wilcoxon ranked-sign test. n = 84 tdTomato, 88 VIVIT
H. Additional replicate of larval infection of *irg1*:*VIVIT-tdTomato*; *kdrl*:*eGFP* and *irg1*:*tdTomato*; *kdrl*:*eGFP* zebrafish (see Figure 4C, 4D). Statistics from Wilcoxon ranked-sign test. n = 109 tdTomato, 108 VIVIT
I. Additional replicate of the TDM injection assay in *irg1*:*VIVIT-tdTomato*; *kdrl*:*eGFP* and *irg1*:*tdTomato*; *kdrl*:*eGFP* zebrafish (see Figure 4E, 4F). Statistics are from Dunn's Kruskal-Wallis multiple comparisons test with Benjamini-Hochberg adjustment for independent tests. 37 tdTomato/IFA, 49 tdTomato/TDM, 25 VIVIT/IFA, 33 VIVIT/TDM
J. Additional replicate of the TDM injection assay in *irg1*:*VIVIT-tdTomato*; *kdrl*:*eGFP* and *irg1*:*tdTomato*; *kdrl*:*eGFP* zebrafish (see Figure 4E, 4F). Statistics are from Dunn's Kruskal-Wallis multiple comparisons test with Benjamini-Hochberg adjustment for independent tests. 86 tdTomato/IFA, 86 tdTomato/TDM, 63 VIVIT/IFA, 62 VIVIT/TDM.

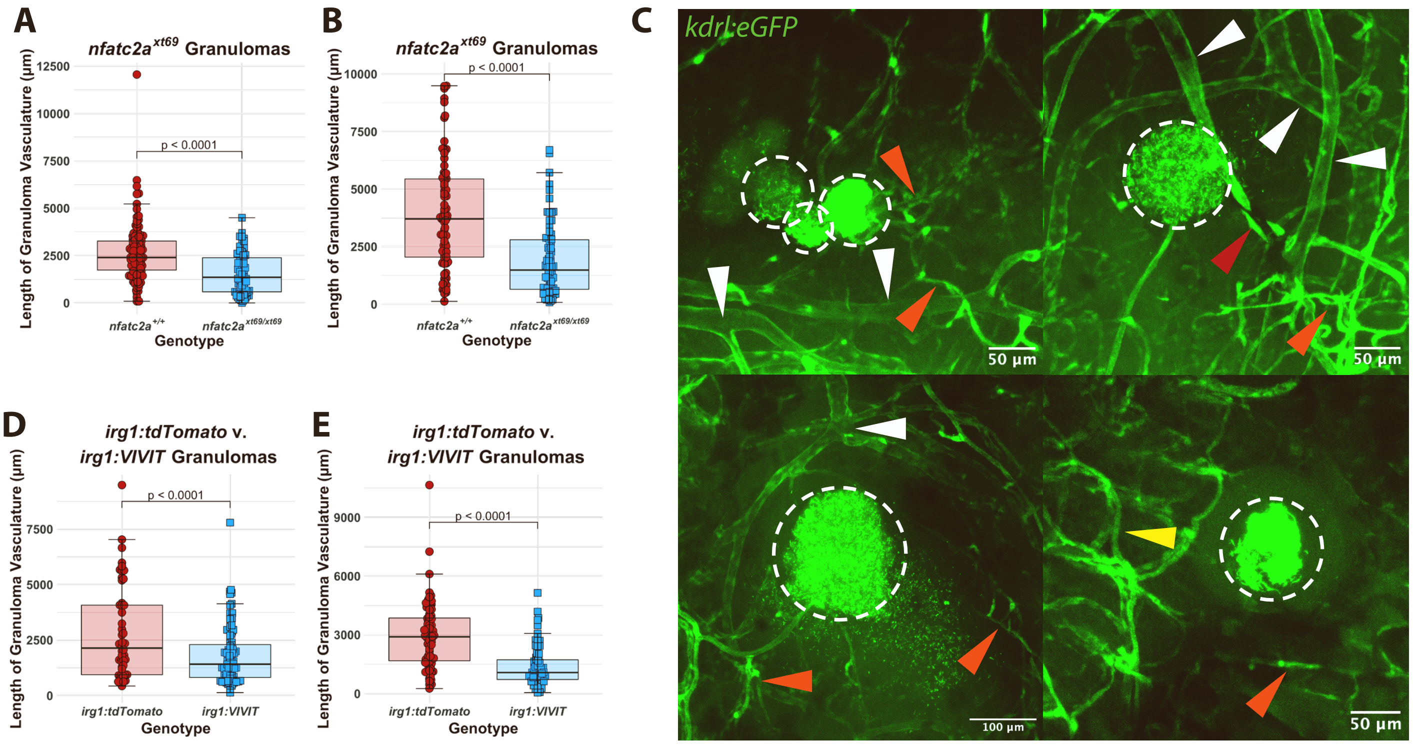
A
nfatc2a xt69 Granulomas
Length of Granuloma Vasculature (µm)
p < 0.0001
nfatc2a +/+
nfatc2a xt69/xt69
Genotype
B
nfatc2a xt69 Granulomas
Length of Granuloma Vasculature (µm)
p < 0.0001
nfatc2a +/+
nfatc2a xt69/xt69
Genotype
C
kdrl:eGFP
50 µm
50 µm
100 µm
50 µm
D
irg1:tdTomato v.
irg1:VIVIT Granulomas
Length of Granuloma Vasculature (µm)
p < 0.0001
irg1:tdTomato
irg1:VIVIT
Genotype
E
irg1:tdTomato v.
irg1:VIVIT Granulomas
Length of Granuloma Vasculature (µm)
p < 0.0001
irg1:tdTomato
irg1:VIVIT
Genotype

Supplemental Figure 3, related to Figure 5. Role for *nfatc2a* and Macrophage NFAT Signaling during Angiogenesis in Adult Granulomas.

A. Additional replicate of the experiment shown in Figure 5B, 5C. *kdrl*:*eGFP*; *nfatc2a*$^{xt69/xt69}$ and *kdrl*:*eGFP*; *nfatc2a*$^{+/+}$ siblings were infected with ~500 CFU *Mm*-tdTomato and sacrified at 18 dpi and then imaged by confocal microscopy. Statistics from Student's t-test. n = 115 wildtype granulomas, 83 homozygous mutant granulomas.
B. Additional replicate of the experiment shown in Figure 5B, 5C. *kdrl*:*eGFP*; *nfatc2a*$^{xt69/xt69}$ and *kdrl*:*eGFP*; *nfatc2a*$^{+/+}$ siblings were infected with ~500 CFU *Mm*-tdTomato and sacrified at 18 dpi and then imaged by confocal microscopy. Statistics from Student's t-test. n = 77 wildtype granulomas, 81 homozygous mutant granulomas.
C. Four images of additional *irg1*:*tdTomato*; *kdrl*:*eGFP* granulomas showing some of the diverse vascular morphologies that can be observed. Dotted circles highlight the necrotic cores of these granulomas. White arrows indicate luminal, mature vessels potentially predating the granuloma. The yellow arrow in the bottom right panel indicates a different type of frequently-seen vascular bed of unknown physiological origin. The dark red arrow indicates vascular damage from a larger vessel. Orange arrows indicate putatively neovascular sprouts and webs, which are likely the major pro-angiogenic effect of these granulomas; however, the diversity of background, also able to be seen in these images, makes true differentiation of these different vessels nearly impossible at any reasonable scale. These images also highlight the diversity of size that can be observed in these granulomas. While some are rather small, others can be dramatically larger.
D. Additional replicate of the experiment shown in Figure 5D, 5E. *irg1*:*VIVIT-tdTomato*; *kdrl*:*eGFP* and *irg1*:*tdTomato*; *kdrl*:*eGFP* fish were infected with ~500 CFU *Mm*-mCerulean and sacrified at 14 dpi and then imaged by confocal microscopy. Statistics from Student's t-test. n = 52 tdTomato, 92 VIVIT
E. Additional replicate of the experiment shown in Figure 5D, 5E. *irg1*:*VIVIT-tdTomato*; *kdrl*:*eGFP* and *irg1*:*tdTomato*; *kdrl*:*eGFP* fish were infected with ~500 CFU *Mm*-mCerulean and sacrified at 14 dpi and then imaged by confocal microscopy. Statistics from Student's t-test. n = 104 tdTomato, 44 VIVIT

A B C D

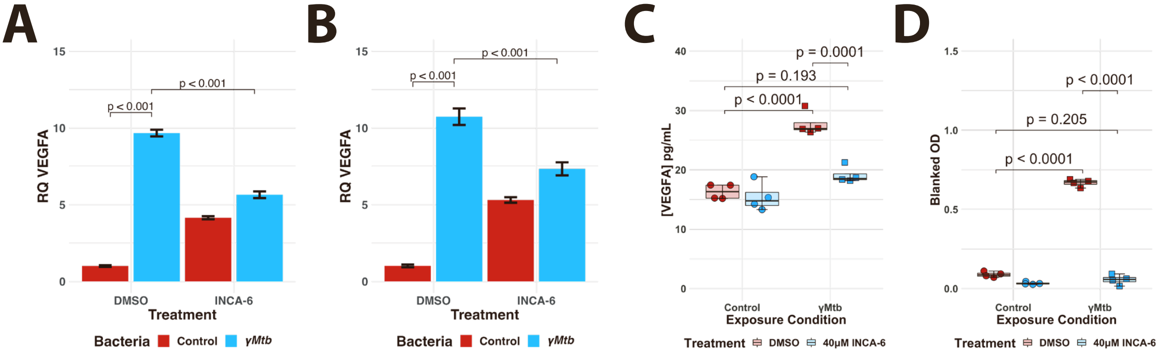

E

	-γMtb	+γMtb	Relationship to VEGFA?
NFATC1	+	++	Modest relationship between nuclear localization and VEGF expression
NFATC2	++	+++	Nuclear localization generally corresponds to VEGF expression
NFATC3	+	++	Weak relationship between nuclear localization and VEGF expression
NFATC4	+/-	+	Expression increased, but not obviously nuclear in most cells

F

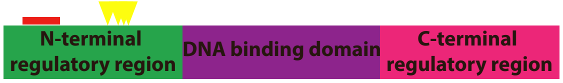

G

```
   1 AAGAGCCGAA TGCACATAAG GTCGCCAGCC CACCCTCCGG ACCCGCATAC CCCGATGATG
  61 TCCTGGACTA TGGCCTCAAG CCATACAGCC CCCTTGCTAG TCTCTCTGGC GAGCCCCCCG
 121 GCCGATTCGG AGAGCCGGAT AGGGTAGGGC CGCAGAAGTT TCTGAGCGCG GCCAAGCCAG
 181 CAGGGGCCTC GGGCCTGAGC CCTCGGATCG AGATCACTCC GTCCCACGAA CTGATCCAGG
 241 CAGTGGGGCC CCTCCGCATG AGAGACGCGG GCCTCCTGGT GGAGCAGCCG CCCCTGGCCG
 301 GGGTGGCCGC CAGCCCGAGG TTCACCCTGC CCGTGCCCGG CTTCGAGGGC TACCGCGAGC
 361 CGCTTTGCTT GAGCCCCGCT AGCAGCGGCT CCTCTGCCAG CTTCATTTCT GACACCTTCT
 421 CCCCCTACAC CTCGCCCTGC GTCTCGCCCA ATAACGGCGG GCCCGACGAC CTGTGTCCGC
 481 AGTTTCAAAA CATCCCTGCT CATTATTCCC CCAGAACCTC GCCAATAATG TCACCTCGAA
 541 CCAGCCTCGC CGAGGACAGC TGCCTGGGCC GCCACTCGCC CGTGCCCCGT CCGGCCTCCC
 601 GCTCCTCATC GCCTGGTGCC AAGCGGAGGC ATTCGTGCGC CGAGGCCTTG GTTGCCCTGC
 661 CGCCCGGAGC CTCACCCCAG CGCTCCCGGA GCCCCTCGCC GCAGCCCTCA TCTCACGTGG
 721 CACCCCAGGA CCACGGCTCC CCGGCTGGGT ACCCCCCTGT GGCTGGCTCT GCCGTGATCA
 781 TGGATGCCCT GAACAGCCTC GCCACGGACT CGCCTTGTGG GATCCCCCCC AAGATGTGGA
 841 AGACCAGCCC TGACCCCTCG CCGGTGTCTG CCGCCCCATC CAAGGCCGGC CTGCCTCGCC
 901 ACATCTACCC GGCCGTGGAG TTCCTGGGGC CCTGCGAGCA GGGCGAGAGG AGAAACTCGG
 961 CTCCAGAATC CATCCTGCTG GTTCCGCCCA CTTGGCCCAA GCCGCTGGTG CCTGCCATTC
1021 CCATCTGCAG
```

H

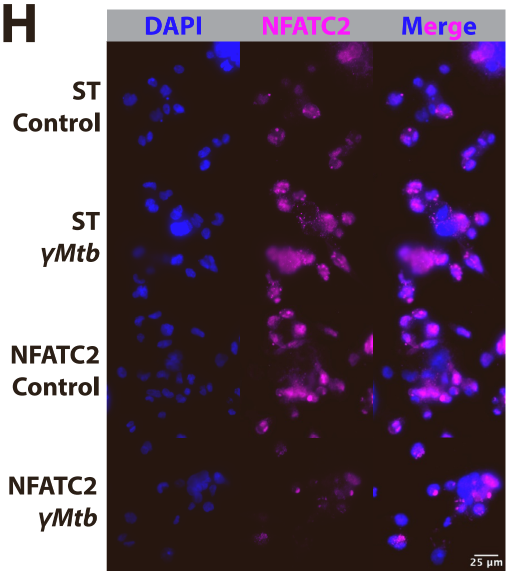

I

sgRNA	Nearest Gene Neighbor (Chromosome)
hU6 ST	CNTN1 (12)
mU6 ST	MTDH (8)
7SK ST	OPN5 (6)
hH1 ST	ENSG00000249941(5)

J

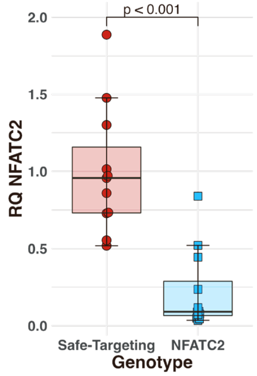

K

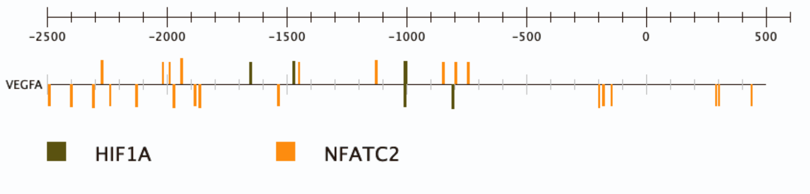

Supplemental Figure 4, Related to Figures 6 and 7. Pharmacological and Genetic Inhibition of NFATc2 Signaling in Human Cells Compromise VEGFA production in Response to γ-irradiated *Mycboacterium tuberculosis.*

A. Additional replicate of the qRT-PCR data shown in Figure 6B. Statistics from ANOVA with Tukey honest significant differences test.
B. Additional replicate of the qRT-PCR data shown in Figure 6B. Statistics from ANOVA with Tukey honest significant differences test.
C. Additional replicate of the ELISA data shown in Figure 6C. Statistics from ANOVA with Tukey honest significant differences test.
D. Additional replicate of the ELISA data shown in Figure 6C. Statistics from ANOVA with Tukey honest significant differences test.
E. Summary of the immunofluorescence screen in A. NFATC2 is present initially and further induced by γ*Mtb* exposure while the others are .
F. Schematic of the structure of NFATC2. Yellow arrows indicate the locations where the sgRNAs target along the gene, all of which are prior to the critical DNA binding domain and are themselves within important protein-protein interaction domains and the nuclear localization regulatory region.
G. Genomic sequence of the second exon of *NFATC2* showing the sequence locations of the four sgRNAs, in yellow.
H. Overlay of NFATC2 and DAPI staining from Fig. 7C. NFATC2 translocates as expected in the ST-transduced cells, but remaining NFATC2 signal is localized entirely to the nucleus in the *NFATC2* sgRNA-transduced cells, potentially due to the relative locations of the antibody (which recognizes an N-terminal epitope) and the sgRNAs (which are concentrated C-terminally to the antibody epitope and the nuclear localization sequence).
I. Table showing the locations of the four safe-targeting sgRNAs. Safe-targeting was chosen to simulate the DNA damage response that occurs during specific sgRNA targeting and the precise sgRNAs were selected from a previously published database of validated sites. The nearest gene neighbor is shown for each gene and the chromosome is listed in parentheses.
J. Transcription of *NFATC2* is reduced after targeting by *NFATC2*-specific sgRNAs from our lentivirus CRISPR/Cas9 approach, suggesting functional knockdown of gene function. Each data point is from an independent isolated clone, statistics by Student's t-test. One point in the ST-targeted group was removed due to aberrantly high NFATC2 expression.
K. Computationally predicted transcription factor binding sites from CiiiDER. Although there are many NFATC2 binding sites in the promoter region of NFATC2, most of them have not yet been ascribed any published functionality.
